# Neural architecture of social punishment: Insights from a queue-jumping scenario

**DOI:** 10.1016/j.isci.2025.111988

**Published:** 2025-02-11

**Authors:** Jiajia Zhu, Xiruo Zhang, Xiaotao Liu, Yan Mu

**Affiliations:** 1 CAS Key Laboratory of Behavioral Science, Institute of Psychology, Chinese Academy of Sciences, Beijing, China; 2 Department of Psychology, University of Chinese Academy of Sciences, Beijing, China; 3 Division of Psychology and Language Sciences, University College London, London, UK; 4 School of Cultures, Languages and Linguistics, The University of Auckland, Auckland, New Zealand

**Keywords:** Neuroscience, Psychology

## Abstract

Punishment in social settings is crucial for maintaining collective interests, yet the underlying mechanisms remain unclear. To address this, we developed a paradigm, the queue-jumping task, where participants imagine experiencing a queue-jumping event through vivid pictorial scenarios. Behavioral findings revealed that individuals prioritized collective interests over personal ones when punishing, highlighting the altruistic nature of social punishment. Neuroimaging results demonstrated that social punishment activated multiple neural circuits associated with social norms (e.g., fusiform gyrus and posterior cingulate cortex), self-related processing (e.g., ventromedial prefrontal cortex and middle cingulate cortex), and punishment implementation (e.g., anterior dorsolateral prefrontal cortex and middle temporal gyrus). Brain network analyses uncovered a social punishment network whose efficacy in information transmission forecasts individuals’ tendency to punish. This study provides valuable insights into the cognitive and neural mechanisms involved in social punishment. The current paradigm closely reflects real-life queue-jumping situations and daily punitive behaviors, demonstrating its generalizability and validity.

## Highlights

• We developed the queue-jumping paradigm to study social punishment
• The paradigm mirrors real-life queue-jumping and correlates with actual punitive behaviors
• Individuals prioritize collective interests, highlighting punishment’s altruistic nature
• Neuroimaging reveals a social punishment network, predicting individual punishment tendencies

## Graphical abstract

**Figure:**
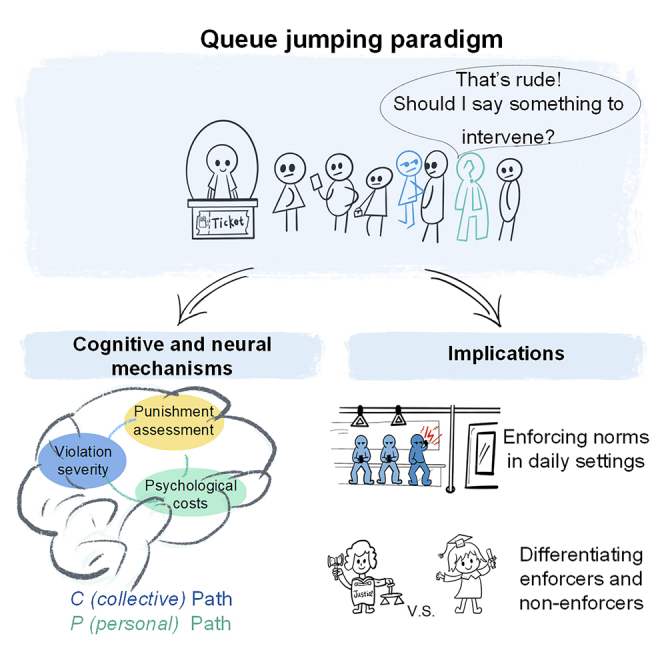

## Introduction

Imagine yourself standing in line at a bustling movie theater, eagerly waiting to purchase your ticket. Suddenly, someone attempts to cut in front of you. Have you ever wondered how you decide whether to confront this individual? What drives and influences our decision to punish the violator? Understanding the mechanisms underlying punishment can aid in making rational and fair decisions in everyday life and judicial sentencing.^1^

Social punishment can be understood as a response to norm violations in order to uphold social norms and safeguard collective interests.^2^^,^^3^^,^^4^ In social contexts like queuing, a deviant behavior (e.g., cutting in line) initially triggers the inclination to enforce the norm (e.g., waiting in line) and may subsequently lead to actions such as verbally reprimanding, thereby maintaining order within the social setting. Humans uniquely possess the capacity to punish on behalf of others and society, not solely for personal benefit, unlike other species.^5^^,^^6^^,^^7^^,^^8^ From infancy and childhood, humans exhibit altruistic punishment tendencies.^9^^,^^10^^,^^11^ Although we may sometimes hesitate to confront rule-breakers due to potential repercussions (e.g., reputational harm),^12^^,^^13^ punishment remains a consistent and essential aspect of human evolution and development.^14^^,^^15^ Social punishment, integral to social justice, fulfills three functions: upholding social norms,^12^^,^^16^ promoting justice,^17^^,^^18^ and fostering cooperation.^19^^,^^20^

Regulating transgression behaviors in society involves a complex interplay of formal and informal systems. Formal systems encompass institutional-level punishments typically enforced through judicial mechanisms,^17^^,^^18^ whereas informal systems rely on non-institutional methods of sanctioning individuals who violate social norms and expectations.^21^^,^^22^ Research on punishment has utilized criminal vignettes, where participants make hypothetical punishment decisions,^23^^,^^24^ and economic games, where participants engage in costly punishment decisions.^25^^,^^26^ In the economic paradigms, for example, participants pay a specified number of tokens to reduce the benefits of the wrongdoer. Although such paradigms effectively quantify punishment, individuals employ diverse forms of punishments in daily situations, such as direct verbal interventions or withholding assistance from wrongdoers.^12^^,^^27^^,^^28^

Consider the real-life example of queue-jumping. Unlike economic paradigms with defined rules and assigned roles (or authorities) to issue fines, no formal system exists for social punishment in these situations. Instead, social punishment, such as disapproval or reprimand, serves to maintain social norms for the majority’s benefits. Such informal and verbal punishments, administered by individuals in everyday life, differ from criminal punishments enforced by legal authorities primarily to achieve justice and ensure public safety. This suggests that social punishment in everyday contexts may operate through different cognitive processes and neural mechanisms than those that have been well validated.^1^^,^^29^^,^^30^ With a growing need to understand altruistic behaviors in daily life,^27^ it is urgent to develop paradigms that closely resemble daily social settings to understand social punishment.

**Figure 1:**
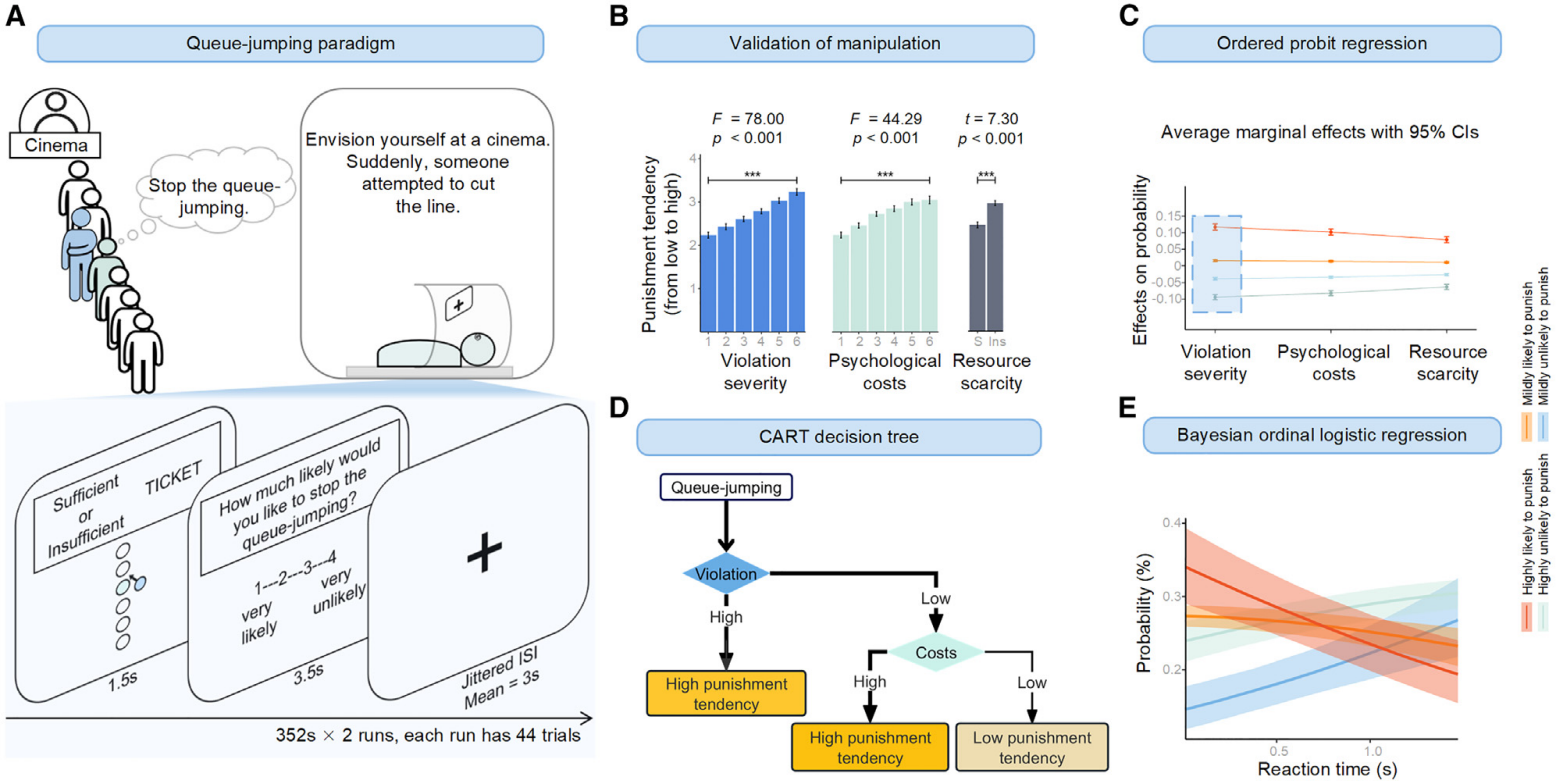
Queue-jumping paradigm and behavioral results (A) A paradigm overview. In the queue-jumping paradigm, participants were instructed to imagine queuing in a line with five other individuals to purchase movie tickets. Six different positions for the queue jumper represented varying levels of norm violation severity. The closer the queue-jumper was positioned to the front of the queue, the more severe the norm violation. Participants’ positions in the queue were used to manipulate six levels of psychological costs. Proximity to the end of the queue resulted in greater psychological costs due to the expected increase in waiting time. We manipulated two levels of remaining tickets: sufficient and insufficient. (B) Paradigm validation. Higher levels of violation severity, psychological costs, and resource scarcity activated an increased tendency among participants to administer punishment compared to lower levels of these conditions (*ps* < 0.001). Data are represented as mean ± SE. (C) Ordered probit regression. With every unit increase in violation severity, psychological costs, and resource scarcity, the likelihood of participants endorsing the highest punishment tendency rose by 12% (being the largest contribution), 10%, and 8%, respectively. Data are represented as mean ± SE. (D) Decision tree. This plot illustrates the cognitive pathways of social punishment, emphasizing the preservation of collective interests intertwined with adherence to norms, and subsequent consideration of self-interests associated with psychological costs. (E) Cumulative ordered logistic regression: the relationship between reaction time and punishment tendency. As reaction times increase, there is a decrease in the inclination to administer punishment, indicating that the simplified cognitive path (from high violation to high punishment tendency) corresponds to the shortest reaction times, thereby eliciting the highest tendency for punishment. The shaded area around the regression line represents the 95% confidence interval. Note: SE, standard error.

To address this gap, we developed a paradigm called the “queue-jumping task” and utilized functional magnetic resonance imaging (fMRI) to examine individuals’ brain activity during this task (Figure 1A). In this paradigm, participants are presented with vivid pictorial scenarios and asked to imagine themselves experiencing a queue-jumping event. This paradigm mirrors a real-life queue-jumping scenario, focusing on a well-established social norm: the first-come, first-served principle.^31^ Punishment has evolved to promote long-term social cooperation, even in one-shot interactions,^14^^,^^32^ which provides a rationale for the tendency to punish in scenarios like queue-jumping. The propensity exists on a continuum, from high to low likelihood, rather than a binary choice of punishing or not.^32^ Consequently, we evaluated participants’ willingness to intervene and prevent a queue-jumper using a 4-point scale (1 = highly unlikely to stop, 4 = highly likely to stop).

Previous research underscores the involvement of the bilateral dorsolateral prefrontal cortex (dlPFC) in the execution of punishment across various paradigms^25^^,^^33^^,^^34^ and neuroimaging techniques methodologies (e.g., fMRI).^26^^,^^35^^,^^36^ These studies have also validated the causal role of dlPFC activity in both facilitating and inhibiting punishment behaviors.^33^^,^^35^^,^^37^ Notably, structural and functional analyses have revealed that the anterior dlPFC, as opposed to the posterior part, is more involved in interactive and context-dependent processes.^38^^,^^39^ The anterior dlPFC has been associated with inferring contextual information during social interactions (e.g., others’ motives and thoughts^40^) and guiding individuals’ behavior (e.g., decision-making^41^^,^^42^). In queue-jumping situations, individuals make social inferences not only about the mental states of others—such as the motivations and thoughts of the jumpers—but also about the expectations of others in the queue and the contextual risks of not getting tickets, which are influenced by the availability of remaining tickets. Given the consistent evidence that the dlPFC and its subregions play a crucial role in both punishment execution and social inference, we anticipate its involvement in making punishment decisions in the queue-jumping task.

Individuals’ punishment decisions are responsive to the severity of the violation.^43^ For example, people show a higher tendency to punish violators who smoke farther from the designated area, indicating a higher degree of violation.^44^ Conversely, another study manipulating the violation’s severity by the size of garbage dropped found no corresponding increase in punishment willingness.^45^ This inconsistency may arise from the challenge of accurately quantifying the degree of violation severity. In queue-jumping situations, as the queue-jumper moves closer to the front, the increasing number of affected queuers signifies a greater breach of social norms.^46^ This paradigm allows for the quantification of norm violations by varying the number of queuers affected. We predict that greater norm violation severity will lead to a higher inclination to punish the violator.

Punishment for norm violations is highly likely to engage neural networks associated with both the emotional and cognitive processes involved in recognizing social violations.^47^^,^^48^ Individuals often experience social aversion when they observe breaches of social norms.^12^ The fusiform gyrus (FG) has been shown to activate in response to these aversive feelings, such as those elicited by illegal parking,^49^ which was further validated by *meta*-fMRI.^50^ Additionally, monitoring and regulating cognitive conflicts between observed violations and established norms may activate the cingulate cortex, a region implicated in conflict monitoring.^51^ Therefore, we anticipate that the brain regions responsive to norm transgressions, such as the FG and cingulate cortex, will be activated when individuals perceive and react to more severe norm violations (i.e., those affecting more queuers).

Psychological cost usually refers to the emotional and cognitive burdens individuals experience when engaging in particular tasks.^52^^,^^53^ In situations where social norms are violated, psychological costs involve negative emotional reactions, such as distress or frustration, along with the cognitive efforts required to process the norm conflicts induced by perceived norm violations and the assessment on personal interests (e.g., impacts on one’s own time, efforts, and entitlement to the resource). Individuals at the end of the queue usually experience longer waiting times and heightened uncertainty, resulting in an increased psychological burden compared to those at the front.^54^ These heightened psychological costs contribute to a stronger inclination to punish.^55^ The increased psychological burden stems from a diminished sense of fairness in resource distribution, thereby negatively impacting personal interests. Additionally, norm-violating behaviors such as cutting in line require additional personal resources, including the time and effort invested in waiting. The queue-jumping paradigm can quantify these psychological costs by manipulating the position of queuers in the line. We hypothesize that participants positioned at the end of the queue, as opposed to the front, will experience higher psychological costs, leading to a greater propensity to punish the violator.

Psychological costs involve self-related processes, such as considerations of personal interests^56^ and neural activity related to self-evaluation, with a focal point on the ventromedial prefrontal cortex (vmPFC).^57^ Moreover, noninvasive manipulations have revealed that vmPFC activation can reliably predict individual punishment tendencies.^58^ Given the role of the vmPFC in self-related evaluation^1^ and norm compliance,^59^ we speculate that higher psychological costs will involve enhanced self-related processes, as indicated by increased vmPFC activity.

Notably, environmental threats, such as resource scarcity, act as distal factors impacting the social norm system and can lead to increased punishment.^60^^,^^61^ Given that resource scarcity may enhance individuals’ propensity to punish, we introduced two levels of resource scarcity in our study: sufficient and insufficient ticket conditions, based on the remaining availability. We aim to explore whether and how resource scarcity influences individuals’ punishment tendencies.

To address these questions, we set up a set of queue-jumping scenarios within the current paradigm: participants wait in a queue of six people to buy movie tickets. Six different positions for the queue-jumper represent varying levels of norm violation severity. The closer the queue-jumper is positioned to the front of the queue, the more severe the norm violation. Participants’ positions in the queue are used to manipulate six levels of psychological costs. Proximity to the end of the queue results in greater psychological costs due to the expected increase in waiting time. The resource scarcity information is conveyed through an icon representing the ticket window, which has two conditions: sufficient remaining tickets and insufficient remaining tickets. First, we hypothesized that participants’ tendency to punish would increase with the severity of violation, the level of psychological costs, and the degree of resource scarcity. Next, we anticipated interactions among these variables. Specifically, we expected that resource scarcity would heighten participants’ sensitivity to both the severity of the violation and the psychological costs, thereby amplifying the impact of these factors on their tendency to punish. We expected a significant interaction between violation severity and psychological costs. In the queue-jumping paradigm, higher violation severity harms the collective interests of the group. When participants punish based on violation severity, they are maintaining the group’s collective interests. In contrast, when punishment is driven by psychological costs, it more reflects a focus on protecting personal interests. Given that individuals have been shown to sacrifice individual interests for collective ones,^19^ it is possible that individuals would similarly prioritize collective over personal interests in the queue-jumping scenarios. Existing research also suggests that individuals are more likely to punish when the outcome benefits them personally and less likely to do so when the punishment conflicts with their own interests.^62^ We hypothesized that participants would be more inclined to punish in the queue-jumping situation when the violation severity is high, regardless of psychological costs. However, when the violation severity is low, the tendency to punish would increase as psychological costs rise.

To uncover the underlying mechanisms involved in social punishment, we employed various analysis techniques. *First*, to explore the significance of evaluating violation severity versus psychological costs, we utilized a machine learning algorithm, Classification And Regression Trees (CART).^63^ This method allows for the ranking of features based on their importance (e.g., da Silva et al.^64^), providing insights into the cognitive mechanisms underlying punishment decisions. *Second*, univariate, multivariate,^65^^,^^66^ and generalized psychophysiological interaction (gPPI) analyses^67^ were used to identify task-related brain regions, neural patterns, and connectivity among these regions. *Third*, high-order functional connectivity (HOFC) was applied, based on topological profiles among brain regions,^68^^,^^69^ offering greater noise resistance and sensitivity than traditional functional connectivity (FC). This approach enhances our ability to predict behavioral performance^68^^,^^69^^,^^70^ and offers a global neural representation of social punishment. *Fourth*, we analyzed the current punishment network’s characteristics (i.e., strength and direction) during both the task and rest states to validate the current punishment network across states. Existing cognitive neuroscience research suggests that the neural systems involved in processing cooperative outcomes—aligned with collective interests—differ from those engaged when assessing personal costs.^71^ Specifically, processing collective interests tends to activate the lateral prefrontal cortex and insula, whereas personal cost assessments primarily engage the ventromedial prefrontal cortex. Therefore, we propose that processing collective versus personal interests may involve distinct brain circuits. Specifically, we hypothesized that brain regions associated with decoding social disgust (e.g., the fusiform gyrus) would be activated in response to recognizing social norm violations, rather than regions linked to value-based aversion (e.g., the anterior insula^1^^,^^25^^,^^26^^,^^72^). Brain regions that process personal interests, such as the vmPFC,^57^^,^^58^^,^^59^ are involved in processing psychological costs. Additionally, we expected that the execution of punishment in queue-jumping scenarios would engage the anterior, but not the posterior, part of the dlPFC.

The current research introduces the queue-jumping paradigm with several advantages. *First*, unlike economic paradigms, the queue-jumping task provides a common daily life situation for quantifying violation severity and psychological costs collectively. This allows for a more precise examination of how individuals weigh these factors when deciding to administer verbal punishment. *Second*, the queue-jumping paradigm differs from hypothetical paradigms using criminal scenarios (e.g., Ginther et al.^73^). While the latter typically requires participants to make hypothetical decisions about moral violations in criminal cases (e.g., murder), the queue-jumping paradigm assesses individuals’ responses to social norm violations that commonly occur in everyday situations. *Third*, the current paradigm helps to deepen our understanding of social punishment by examining both the psychological costs and the influence of social factors like resource scarcity.

Notably, to verify the ecological validity and generalizability of the queue-jumping paradigm, we conducted a series of supplementary studies and examined whether individuals’ punishment tendencies can predict their actual punishment behaviors in real-life situations (see Supplementary Studies 1–4 for details). Specifically, we compared punishment tendencies across various paradigms, including economic, criminal, and social norm scenarios. The goal was to examine whether and how these tendencies predict participants’ real-world social punishment behaviors, such as the frequency with which they intervene to prevent social norm violations in daily life and how they respond to real-life instances of queue-jumping or other norm violations. Given that the current paradigm mirrors a real-world situation and formal punishment is more institutionalized and typically determined by legal statutes or formal rules,^74^ we hypothesized that the punishment tendency in the queue-jumping paradigm is more directly linked to daily social punishment behaviors, as opposed to the more formal, system-driven punishments that dominate the criminal and economic paradigms.

## Results

### Paradigm validation: Behavioral level

We calculated the mean punishment tendency scores of participants across varying levels of violation severity, psychological costs, and resource scarcity. As expected, repeated measures analysis of variance (ANOVA) and paired sample t tests showed higher levels of violation severity (*F*_(1.85, 105.31)_ = 78.00, *p*_Greenhouse - Geisser correction_ < 0.001, *η*^2^_partial_ = 0.58; Greenhouse–Geisser corrected F test results were reported; the same below), psychological costs (*F*_(2.30, 131.15)_ = 44.29, *p* _Greenhouse–Geisser correction_ < 0.001, *η*^2^_partial_ = 0.44), and resource scarcity (*t*_(58)_ = 7.30, *p* < 0.001, Cohen’s d = 0.95) led to an increased punishment tendency compared to lower levels of these conditions (Figure 1B; post-hoc comparisons in Table S1). Regression analyses further confirmed the effects of these factors and revealed an interaction between violation severity and resource scarcity, indicating that resource scarcity amplifies participants’ sensitivity to violations (see the supplemental information on logistic regression).

Next, we examined which of the three factors—violation severity, psychological costs, and resource scarcity—exert the most substantial influence on participants’ punishment tendencies. Previous research has suggested that individuals’ psychological states while queuing often display a *nonlinear* relationship with changes in their positions,^53^ a phenomenon commonly referred to as the marginal effect. To assess these marginal effects, we utilized an ordered probit regression, a statistical method well suited for fitting ordered categorical data.^75^ The regression analysis revealed that the three factors had incrementally increasing effects on participants’ punishment tendencies (see the supplemental information on ordered probit regression). Specifically, for each unit increase in violation severity, psychological costs, and resource scarcity, the probability of participants exhibiting the highest punishment tendency increased by 12%, 10%, and 8%, respectively (Figure 1C; Table S2). These results underscored the predominant role of violation severity (with a 12% increase, the most significant contribution) in shaping punishment tendencies.

To elucidate the cognitive pathway involved in social punishment, we conducted a decision tree analysis using R package *rpart*.^63^ We randomly assigned 70% of responses to the training set and retained 30% as the test set. For model construction, we applied 10-fold cross-validation with a cost-complexity parameter of 0.001.^63^ We classified the initial three levels as low and the subsequent three as high for violations (or costs; verification of this classification in Figure S1A). Based on the probabilities of the four punishment ratings, we encoded the ratings of “mildly likely” or “highly likely” to stop queue-jumping as indicating a high punishment tendency, whereas the other two “unlikely” ratings were classified as a low punishment tendency. As demonstrated in Figure 1D, participants bypassed the assessment of psychological costs, exhibiting a heightened punishment tendency in high violation conditions. Conversely, in low violation conditions, participants engaged in a more thorough evaluation of psychological costs, displaying a high punishment tendency at high-cost levels and a low punishment tendency at low-cost levels. The model, which achieved a prediction accuracy of 0.67 (95% confidence interval [CI] = 0.65, 0.70), suggests that participants prioritize norm maintenance as their primary concern when making such punishment decisions, followed by the protection of personal interests. Resource scarcity did not significantly impact this outcome (Figure S1B and the supplemental information on decision tree analyses). Consistently, a follow-up cumulative ordered logistic regression using the Bayesian framework (employing the R package *brms*^76^) revealed that for each unit increase in reaction times (RTs), there was a corresponding decrease in the log-odds of moving to a higher category of the ordinal rating variable (estimate = −0.52, 95% CI: −0.75, −0.31, Rhat = 1.00; Figure 1E; Table S3). This implies that as RTs increase, the punishment tendency decreases (see the supplemental information on punishment tendency and RTs). This finding further supports the idea that the path with the longest RTs (low violation severity → low psychological costs) results in the lowest tendency for punishment.

### Paradigm validation: Neural level

**Figure 2:**
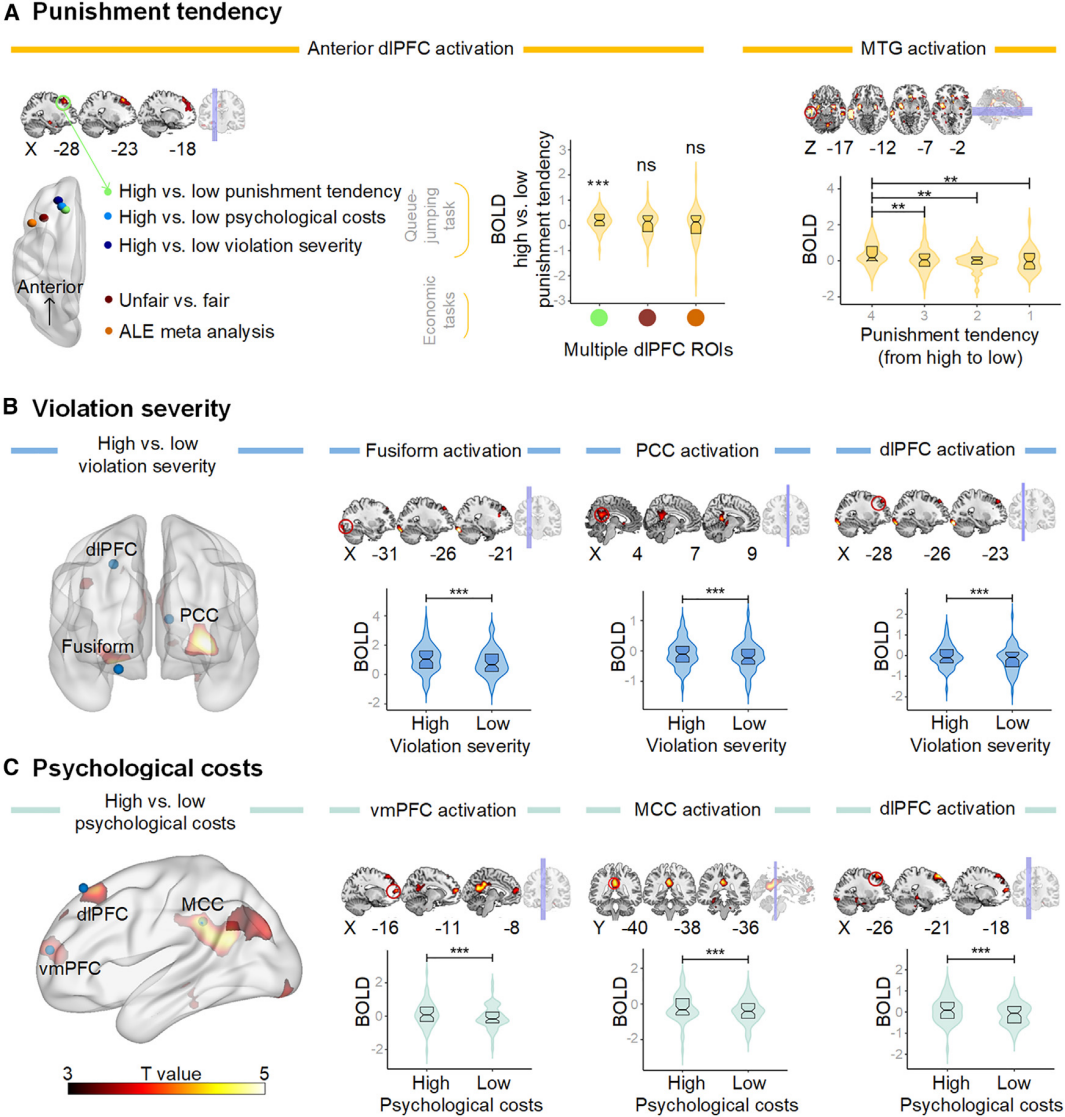
fMRI results (A) Neural activity of punishment tendency. The left plot depicts the activation of the social dorsolateral prefrontal cortex (dLPFC) in the current paradigm, located near the anterior part (BA 9), whereas economic dLPFC activation in previous studies consistently resides in the posterior segment of the dLPFC (BA 44/46). *The middle plot* illustrates increased activation in the left dorsolateral prefrontal cortex (dlPFC) in the contrast between high and low punishment tendencies. One sample t test (compared to zero). *The right plot* illustrates the activation of the left middle frontal gyrus (MTG) in processing different levels of punishment. Note: unfair vs. fair: [-36 25 42],^26^ BA 46. ALE meta-analysis: [-46 20 40],^72^ BA 44. The above coordinates are based on the MNI (Montreal Neurological Institute) coordinate system. (B) Neural activity of violation severity. The left plot illustrates increased activation in the left fusiform, the right posterior cingulate cortex (PCC), and the left dlPFC in the contrast between high and low violation severity. The violin plots describe BOLD (blood oxygenation level-dependent) signals of the left fusiform, the right PCC, and the left dlPFC in the conditions of high and low violation severity. (C) Neural activity of psychological costs. The *left plot* depicts increased activation in the left ventromedial prefrontal cortex (vmPFC), the left middle cingulate cortex (MCC), and the left dlPFC. The violin plots illustrate BOLD signals of the left vmPFC, the right MCC, and the left dlPFC in the conditions of high and low psychological cost. Note: ^∗∗^*p* < 0.01, ^∗∗∗^*p* < 0.001, ns: no significant difference. In the boxplot, the upper edge represents the third quartile, whereas the lower edge indicates the first quartile. The horizontal line within the box denotes the median.

To validate the paradigm at the neural level, we first classified trials rated as “mildly or highly likely” to punish as the high punishment condition, whereas those rated as “mildly or highly unlikely” were categorized as the low punishment condition. The contrast analysis between high and low punishment conditions revealed heightened activation in the left dlPFC (x = −18, y = 33, z = 54, *p*_FWE correction_ = 0.007, *t* = 4.16, k = 216; Figure 2A). These results are consistent with previous research.^1^^,^^30^

To explore whether the dlPFC activation in our paradigm differs from that in previous economic and criminal punishment, we employed region of interest (ROI) analysis using the two sets of coordinates: BA 44 (x = −46, y = 20, z = 40), representing the main effect of social punishment measured using economic or criminal paradigms from an activation likelihood estimation (ALE) meta-analysis,^72^ and BA46 (x = −36, y = 25, z = 42), indicating the contrast of unfair vs. fair.^26^ The dlPFC in our paradigm is situated near the anterior region (BA 9). There was no significant activation difference between high and low punishment conditions in the two previous ROIs identified activation in the posterior segment: *t*_*(*55) meta_ = 0.52*, p* = 0.60, Cohen’s d = 0.07; *t*_*(*55) unfair_ = 1.69, *p* = 0.10, Cohen’s d = 0.23.

Using a parametric general linear model (GLM) analysis, we further investigated how brain activity varied with different levels of punishment tendency, from “highly unlikely” to “highly likely” to punish. The results indicated that participants engaged the left middle frontal gyrus (MTG; x = −57, y = −30, z = −15, *p*_FWE_ = 0.04, *F* = 9.43, k = 110), in response to varying levels of punishment tendency, with the highest activation observed for the most severe punishment tendency (Figure 2A). Additionally, positive correlations were observed between the activation levels of the MTG and dlPFC regions (0.32 < *rs*_(56)_ *<* 0.55, *ps* < 0.05; Figure S2A).

To assess average and pattern differences in neural activations associated with varying levels of violation severity, we employed both univariate and multivariate analyses. The univariate analysis revealed heightened activation in the left fusiform gyrus when contrasting high versus low levels of violation severity (x = −21, y = −90, z = −18, *p*_FWE_ = 0.03, *t* = 5.67, k = 135, Figure 2B). Furthermore, increased activation was observed in the right posterior cingulate cortex (PCC) (x = 33, y = −96, z = −6, *p*_FWE_ = 0.02, *t* = 5.53, k = 144) and the left dlPFC when contrasting high versus low violation conditions (x = −24, y = 42, z = 45, *p*_FWE_ = 0.003, *t* = 3.75, k = 33, Table S4). There was a correlation between the activation levels of the PCC and dlPFC regions (*rs* _(59)_ *=* 0.36, *p* = 0.005, Figure S2B). MVPA results indicated that the left cerebellum could differentiate between high and low levels of violations (x = −9, y = −66, z = −21, *p* _FWE_ < 0.001, *t* = 4.23, Z_k_ = 3.93, Table S5). This finding is consistent with previous research highlighting the cerebellum’s involvement in norm-related processing.^77^^,^^78^

To assess average and pattern differences in neural activity associated with varying levels of psychological costs, we conducted both univariate and MVPA analyses (Figure 2C). The univariate analysis revealed increased activation in the left middle cingulate cortex (x = −3, y = −39, z = 33, *p*_FWE_ < 0.001, *t* = 4.95, k = 469) and the ventromedial prefrontal cortex when contrasting high versus low levels of psychological costs (x = −12, y = 57, z = 15, *p*_FWE_ = 0.04, *t* = 4.37, k = 136; Table S6). Both regions are known to be involved in self-referential processing.^1^^,^^57^ Additionally, there was heightened activation in the left dlPFC when contrasting high versus low psychological costs (x = −21, y = 36, z = 54, *p*_FWE_ = 0.02, *t* = 4.83, k = 160). The activation intensity of the left dlPFC was positively correlated with that of the vmPFC and MCC regions (0.55 < *rs*_(59)_ *<* 0.68, *ps* < 0.001; Figure S2C). However, MVPA analyses did not show significant activity when contrasting high versus low psychological costs.

We used univariate and multivariate analyses to investigate the neural mechanisms underlying the processing of resource insufficiency. Results suggested that participants make more inferences about the intentions of others in the queue (e.g., the left temporo-parietal junction; x = −57, y = −63, z = 27, *p*_FWE_ = 0.002, *t* = 3.06, k = 38, angular gyrus part, small volume correction) and exhibit a stronger desire to conform to social norms (e.g., the right caudate; x = 15, y = −9, z = 27, *p*_FWE_ < 0.001, *t* = 5.76, k = 5.10) when resources are insufficient compared to sufficient (see the supplemental information on neural results of resource scarcity).

### Constructing the neural network of social punishment

**Figure 3:**
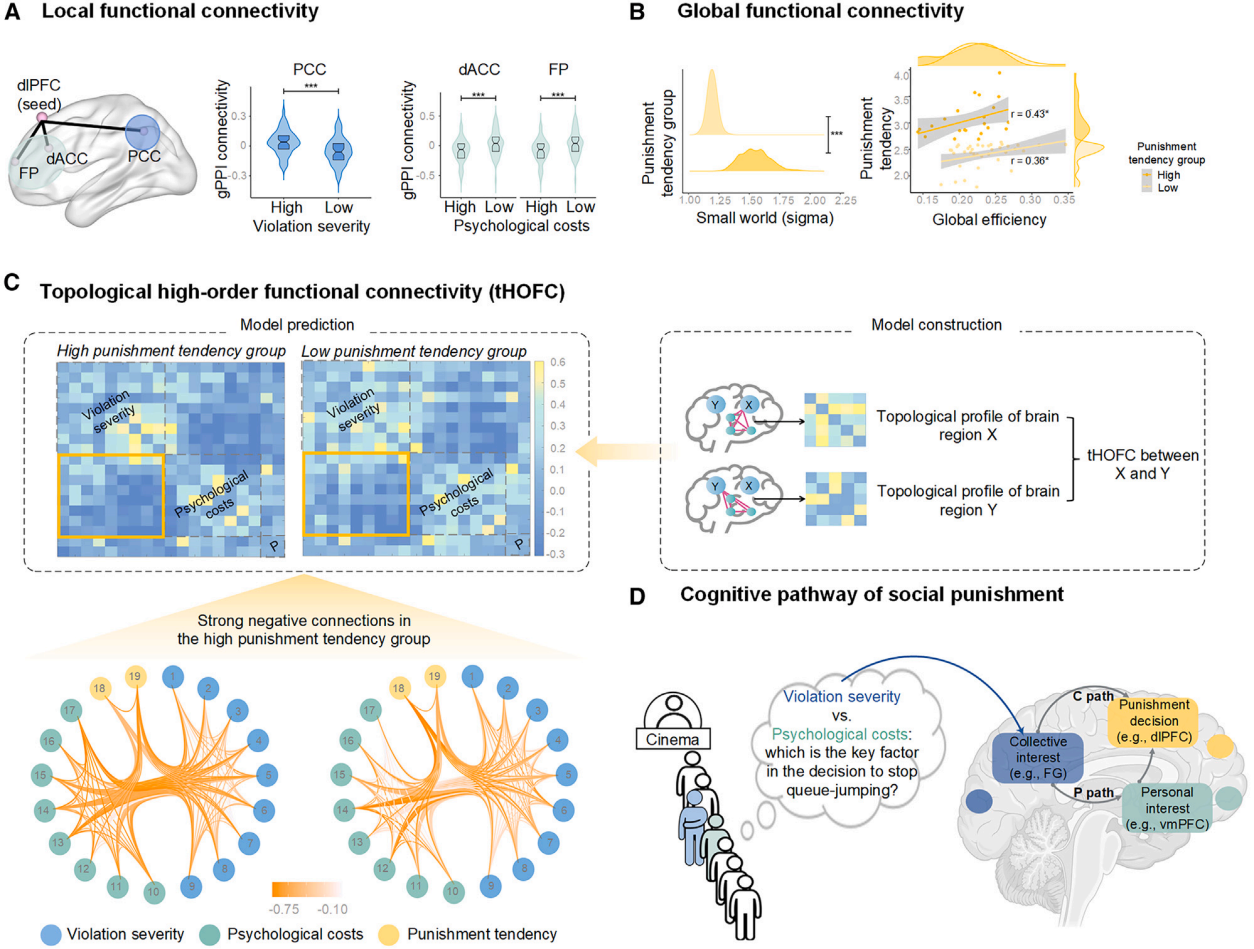
Constructing the neural network of social punishment (A) Local functional connectivity. The *left plot* depicts the gPPI connections seeded in the left dlPFC. The *middle plot* illustrates increased gPPI coupling between the left dorsolateral prefrontal cortex (dlPFC) and the left dorsal posterior cingulate cortex (PCC) when contrasting conditions with high and low violation levels. The *right plot* illustrates increased gPPI coupling between the left dlPFC and the left frontal pole (FP) as well as the left dorsal anterior cingulate cortex (dACC) in the contrast of low vs. high levels of psychological costs. In the boxplot, the upper edge represents the third quartile, whereas the lower edge indicates the first quartile. The horizontal line within the box denotes the median. Note. ^∗∗∗^*p* < 0.001. (B) Global functional connectivity. The left plot: the social punishment network exhibited a higher small-world attribute in the participants with high punishment tendency compared to those with low punishment tendencies. The right plot: the global efficiency of the social punishment network could positively predict punishment tendency in both high and low punishment tendency groups. The shaded area around the regression line represents the 95% confidence interval. Note. ^∗^*p* < 0.05, ^∗∗∗^*p* < 0.001. (C) Topological high-order functional connectivity (tHOFC). The *right top* plots show the construction process of tHOFC. The *left top and the left bottom* depict the performance of the tHOFC model. tHOFC could predict the punishment tendency of participants in the task-based brain network (AUC = 0.58, ACC = 61.02%). Compared with the low punishment tendency group, participants in the high punishment willingness group exhibited stronger positive functional connections between brain regions involved in processing norm violation severity. Additionally, there were stronger negative connections between brain regions processing violation severity and degrees of psychological costs. The numbers 1 to 19 indicate the 19 nodes comprising the punishment brain network; see details in Table S9. (D) A framework for understanding social punishment processing. Individuals tend to focus first on the impact of violations on the majority of the group rather than on themselves when making punishment decisions for observed violations of social norms. High severity of norm violation causes a high impact on collective interests. The social punishment decision for high severity of norm violation follows the *C* (collective) path, which preserves the collective interest. In contrast, the low severity of norm violation causes a low impact on collective interests. The penalty for a low severity of norm violation follows the *P* (personal) path, which protects personal interest. The *C* path involves brain regions related to norm processing (e.g., the fusiform gyrus [FG], posterior cingulate cortex [PCC]). Enhanced functional connections between these brain regions lead to individuals’ high tendency to punish. The *P* path processes personal interest loss, which involves brain regions such as the ventromedial prefrontal cortex (vmPFC) and middle cingulate cortex (MCC). Functional connections between these brain regions are enhanced when personal interest loss is high, resulting in a high tendency to punish. The left anterior dorsolateral prefrontal cortex (dlPFC) and the left middle temporal gyrus (MTG) drive individuals' punishment decisions. Moreover, individuals with a high punishment tendency demonstrate stronger internal connectivity in brain regions related to the *C* path and weaker internal connectivity in brain regions associated with the *P* path, revealing the altruistic nature of social punishment (This figure was created with BioRender.com).

To construct the neural network associated with social punishment, we investigated the local functional connectivity among brain regions activated during the task using generalized psychophysiological interaction (gPPI) analysis.^67^ Since the left dlPFC was activated when contrasting high versus low punishment conditions, we designated this region as the seed for examining its interactions with other brain areas across the whole-brain scale. The gPPI analysis revealed increased coupling between the left dlPFC and the left dorsal PCC during the high versus low violation conditions (x = −15, y = −54, z = 42, *p*_FWE_ = 0.05, *t* = 4.34, k = 256; Figure 3A; Table S7). Additionally, stronger connectivity was observed between the left dlPFC and the left frontal pole (FP) (x = −15, y = 60, z = 15, *p*_FWE_ = 0.01, *t* = 4.37, k = 50), as well as the left dorsal anterior cingulate cortex (dACC) (x = −9, y = 30, z = 27, *p*_FWE_ = 0.01, *t* = 4.13, k = 55), when contrasting low versus high levels of psychological costs (Figure 3A). Moreover, gPPI analyses indicated increased connectivity between the brain regions related to psychological costs and the frontal cortex, including the inferior frontal gyrus (IFG) and orbitofrontal cortex (OFC) (Table S7).

We further investigated whether task-related brain regions, which are spatially distributed across different parts of the brain, form an *effective* yet *distinct* neural network specifically involved in punishment decision-making. We first constructed a punishment network comprising 19 nodes (Table S8) identified through GLM, MVPA, and gPPI analyses. Graph theory analysis revealed that this punishment network exhibits small-world characteristics, indicating efficient and rapid information transfer among the nodes (*t*_(58)_ = 5.44, *p* < 0.001, Cohen’s d = 0.71, one-tailed). Subsequently, participants were categorized into high and low punishment tendency groups based on their average punishment tendency (mean and SD values provided in Table S9). The analysis showed that the social punishment network had a higher small-world attribute in the high punishment tendency group compared to the low punishment tendency group (*t*_(56)_ = 3.10, *p* = 0.003, Cohen’s d = 0.84, two-tailed, Figure 3B). Additionally, the global efficiency of the social punishment network was positively associated with a high punishment tendency in both groups (Figure 3B).

In our network analysis utilizing topological high-order functional connectivity (tHOFC) through BrainNetClass^69^ (Table S10), connection coefficients (i.e., functional connections based on topological profiles) were used as features for classifying punishment tendency groups. We applied LASSO (least absolute shrinkage and selection operator) regression (λ = 0.05) to select contributing edges. The model’s performance was evaluated using LOOCV (leave-one-out cross-validation). The tHOFC results showed that connection coefficients could predict individual punishment tendency within the task-based brain network (AUC = 0.58, ACC = 61.02%). However, the current punishment network in the resting-state mode failed to distinguish between the high and low punishment groups. Specifically, tHOFC analysis revealed that participants in the high punishment group exhibited stronger positive functional connections between brain regions involved in processing violation severity compared to those in the low punishment group. Additionally, there were stronger negative connections between brain regions responsible for violation severity and those involved in processing psychological costs (Figure 3C). These findings suggest that when brain regions evaluating the severity of violations are active, regions responsible for processing psychological costs may be inhibited.

### Ecological validity and generalizability of the queue-jumping paradigm

#### Supplementary Study 1

To examine whether participants’ tendency to punish in the queue-jumping paradigm would be correlated with the frequency of their social punishment behaviors in everyday life, we conducted a pre-registered Supplementary Study 1 (*N* = 247; see Methods of Supplementary Study 1 section for details). We used a similar set of queue-jumping scenarios as those used in the fMRI study to assess participants’ punishment tendencies. ANOVA results replicated the effects of violation severity and psychological costs on punishment tendency (*F*_violation severity (1, 246)_ = 422.63, *p* < 0.001, *η*^*2*^_*partial*_ = 0.63; *F*_psychological costs (1, 246)_ = 331.76, *p* < 0.001, *η*^*2*^_*partial*_ = 0.57; see ANOVA and post-hoc results in Tables S11 and S12).

Additionally, we asked participants to rank a set of representative scenarios from 1 to 9 based on their perceived severity and cost levels. Lower ranking scores represent higher levels of severity and costs. ANOVA results revealed that participants rated queue-jumping behavior as most severe when it occurred at the front positions of the queue, in contrast to the middle or bottom ones (ranking scores from front to bottom: 3.14, 4.92, and 6.95, *F*_(2, 492)_ = 565.15, *p* < 0.001, *η*^*2*^_*partial*_ = 0.70; Table S13). Moreover, they reported more psychological costs when they were placed at the bottom, compared to when they were placed in the middle or at the front (ranking scores from bottom to front: 4.26, 5.01, and 5.74, *F*_(2, 492)_ = 152.70, *p* < 0.001, *η*^*2*^_*partial*_ = 0.38; Table S13). The findings further validate the effective manipulation of the two factors.

We assessed various types of reasons influencing punishment decisions using a 7-point scale (from 1 = *strongly disagree* to 7 = *strongly agree*) across domains: social norm violation, increased waiting time, affecting ticket purchase, affecting others’ interests, causing chaos, and violating personal norms. ANOVA results indicated significant differences among these factors (*F*_(5, 1230)_ = 11.87, *p* < 0.001, *η*^*2*^_*partial*_ = 0.05), with participants showing stronger agreement on two reasons—social norm violation and increased waiting time—compared to the other factors (post-hoc comparisons in Table S14).

We assessed participants’ tendency to punish in queue-jumping scenarios as well as other paradigms, such as criminal scenarios,^24^ economic punishment,^26^ and violation of social norms,^22^ and measured the frequency of their social punishment behaviors (i.e., punishing social violations) in everyday life.^79^ There was a significant correlation between punishment tendency in the queue-jumping paradigm and the frequency of their social punishment behaviors in everyday life (*r*_queue_ = 0.31, *p* < 0.001); however, punishment in other paradigms demonstrated weak and no correlations with this frequency (Table S15).

#### Supplementary Study 2

To further justify the ecological validity and generalizability of the queue-jumping paradigm, we conducted a pre-registered Supplementary Study 2 (*N* = 194; see Methods of Supplementary Study 1 section for details). We first asked participants to evaluate how easily the paradigm evoked participants’ recollections of real-life queue-jumping scenarios on a seven-point Likert scale (1 = very difficult to 7 = very easy). The average rating was 5.51 (±1.24), with no participants selecting “very difficult,” suggesting that the paradigm effectively prompts associations with real-life queue-jumping experiences. Consistent with Supplementary Study 1, we also included a variety of punishment indicators. In addition, to assess participants’ actual punishment practices in everyday situations, we adapted a study using a diary method to measure punishment behaviors in response to social norm violations.^12^ Please refer to Methods of Supplementary Study 2 section for details section for details.

Consistent with Supplementary Study 1, we found significant correlations between punishment tendency in the queue-jumping paradigm and the frequency of punishing violation behaviors in everyday life (*r* = 0.31, *p* < 0.001) as well as their actual social punishment practices (*r*_real queue-jumping_ = 0.37, *p* < 0.001; *r*_real norm violation_ = 0.34, *p* < 0.001); however, participants’ punishment performance in previous paradigms had little to no predictive ability for their punishment frequency in daily life (*r*_criminal paradigm_ = −0.21, *p* = 0.003; *r*_social norm violation paradigm_ = 0.05, *p* = 0.46; *r*_economic paradigm_ = 0.02, *p* = 0.78) as well as their actual punishment practices in the real queue-jumping (*r* _criminal paradigm_ = −0.07, *p* = 0.34; *r*_social norm violation paradigm_ = 0.17, *p* = 0.02; *r*_economic paradigm_ = 0.02, *p* = 0.79) and the recent social norm violation cases (*r*_criminal paradigm_ = −0.16, *p* = 0.03; *r* _social norm violation paradigm_ = 0.11, *p* = 0.15; *r*_economic paradigm_ = 0.01, *p* = 0.92). These results suggest that individuals’ punishment tendencies in the current paradigm are more closely linked to real-life punitive actions than their performance in other punishment paradigms.

#### Supplementary Study 3

To further validate the generalizability of the queue-jumping paradigm, we conducted a pre-registered Supplementary Study 3, investigating whether participants' punishment tendencies varied based on their professional backgrounds. Specifically, we explored if individuals with law backgrounds exhibited a higher propensity for punishment compared to those from non-law backgrounds (86 participants with law backgrounds, 102 without; see Methods of Supplementary Study 3 section for details). The results indicated that the punishment tendency in the queue-jumping paradigm could effectively differentiate the two groups, with the law group showing a lower tendency to punish compared to the non-law group (law vs. non-law: 4.01 ± 0.95 vs. 4.40 ± 0.76; *t* = −3.08, *p* = 0.002). It may be due the fact that individuals with law backgrounds are more likely to rely on objective criteria such as focusing on the illegality of the violation itself rather than on the harm.^80^ Similarly, professional medical workers have been proven to show a disregard for the patient’s pain in the absence of clear medical evidence.^81^

#### Supplementary Study 4

Given that real-life punishment is a binary choice (i.e., punishing or not), we used a within-subjects design to test the relationship between the four-point punishment tendency rating scale and the binary punishment decision in Supplementary Study 4 (*N* = 32; see Methods of Supplementary Study 4 section for details). The results showed no significant difference between the frequency of participants choosing punishment in the binary task and the combined frequency of selecting 3 (“mildly likely to stop”) and 4 (“highly likely to stop”) in the four-point task (binary vs. combined: 45% ± 0.27 vs. 48% ± 0.28; *t* = −1.40, *p* = 0.17). Furthermore, a correlation analysis showed a significant correlation between participants’ tendencies in the binary task and their tendencies in the four-point task (*r* = 0.32, *p* < 0.001).

## Discussion

This study introduces the queue-jumping paradigm to elucidate the cognitive and neural mechanisms underlying punishment decision-making within everyday contexts. The behavioral and neural findings validate this queue-jumping paradigm, enhancing our understanding of the cognitive processes and neural mechanisms involved in social punishment. The regression results indicate that participants prioritize the assessment of collective over personal interests when administering punishment. Decision tree and RT analyses jointly show that higher violation severity leads to firm and swift punishment decisions, underscoring the importance of prompt responses to significant norm breaches. Furthermore, fMRI results reveal that social punishment engages multiple neural circuits associated. These include regions associated with social norms (e.g., fusiform gyrus and PCC), self-related processing (e.g., vmPFC and MCC), and punishment execution (e.g., anterior dlPFC and MTG). Brain network analyses confirm the close relationship among these regions within the current punishment network, effectively predicting individuals’ punishment tendencies.

### Return to daily: The queue-jumping paradigm

While punishment in non-human animals primarily serves self and familial interests (e.g., Scalefin Anthias^82^ and chimpanzees^8^), human punishment plays a crucial role in maintaining social norms and fostering cooperation.^8^^,^^82^^,^^83^ Economic punishment paradigms, which focus on financial costs and consequences, offer advantages for understanding punishment in controlled settings with quantifiable data. However, these paradigms often conflate selfish motivations with punishment actions, making it difficult to isolate self-interest.^48^^,^^84^ Some researchers question the self-interest hypothesis, highlighting instances where individuals deviate from self-interest maximization.^85^ Considering these debates on economic paradigms and the context-dependent nature of punishment decisions, the current paradigm manipulates levels of violation severity and psychological costs, offering a realistic daily situation to comprehend punishment intentions that require balancing individual against collective interests. Additionally, contextual factors, such as competitive versus cooperative situations, can elicit self-regarding behavior.^86^ The results of the supplementary studies collectively suggest that the punishment tendency in the queue-jumping paradigm could predict participants’ actual punishment behaviors in their daily lives. More importantly, individuals’ punishment tendencies shown in the current paradigm relative to their punishment performance in the other punishment paradigms are more uniquely and tightly linked to real-life punitive actions, thereby supporting the ecological validity and generalizability of the paradigm.

### Collective versus personal interests: Unraveling the cognitive dynamics of social punishment

Consistent with previous literature on social punishment (e.g., Kessler et al.^44^ and Rucker et al.^43^), our study reveals that individuals’ punishment tendencies increase with the extent of the violation impacting more individuals in the queue. In line with Efrat-Treister et al.’s findings,^54^ our results indicate that greater psychological costs predict a higher tendency to punish. More importantly, the probit regression results reveal that norm severity has the highest marginal effect, followed by psychological costs and resource scarcity. These findings suggest that people prioritize evaluating pronounced violations over factors related to their personal interests, providing clear evidence of the altruistic nature of punishment in the queuing context. Additionally, the decision tree analysis supports the notion of altruistic punishment, with the severity of the violation prioritized at the initial decision node, regardless of resource scarcity.

Individuals possess an innate system for upholding collective interests.^87^^,^^88^ Notably, our findings suggest that high and low levels of violation severity prompt distinct cognitive processing pathways, as revealed by the decision tree analysis. Participants bypassed the consideration of psychological costs and exhibited shorter reaction times under high levels of violation severity, indicating a simpler cognitive process in punishment decision-making. This heightened readiness to punish prioritizes collective interests over personal concerns, aligning with previous research on spontaneous punishment.^89^ Similarly, neuroimage research reveals that efficient connectivity between brain regions serves as a crucial indicator of rapid cognitive processing and evolutionary advantage in humans.^90^

### Decoding justice: Common yet distinct neural mechanisms underlying social punishment

At the neural level, the activation of the left fusiform gyrus and right PCC in the contrast between high and low violation conditions suggests a blend of emotional and cognitive processing when addressing breaches of social norms during punishment decision-making. Previous studies have found that individuals with elevated social anxiety exhibit greater fusiform gyrus activation when witnessing norm violations in everyday contexts, indicating heightened negative emotions in response to such behaviors.^22^ Moreover, recent meta-analyses of fMRI research has underscored the fusiform’s pivotal role in representing generalized social disgust.^50^ Therefore, the fusiform activation observed in the current study reflects social aversion to norm violations. In contrast to the anterior insula’s (AI) involvement in aversive reactions to economic violations, like unfairness,^1^^,^^25^^,^^26^^,^^72^ it seems that the fusiform gyrus serves as a key foundation for detecting norm violations in everyday social scenarios, particularly concerning emotional processing.

Norm breaches often undermine established social values, posing threats to the existing normative system and personal norms.^91^ Previous research has shown that the cingulate cortex is generally involved in conflict monitoring, such as discrepancies between expected fairness and actual unfairness.^25^^,^^26^ The PCC, a central hub within the default network, has demonstrated its role in signaling a prevention focus (i.e., a buffer against stereotype threats).^92^ Increased PCC activation suggests that people prioritize social duties and obligations (an outward focus) over self-processing (an inward focus) in threatening situations.^92^^,^^93^^,^^94^ Moreover, neural activity in this area indicates the monitoring of changes in rules and adjusting subsequent behavior to align with norms.^95^^,^^96^ Additionally, the gPPI analysis revealed positive functional connectivity between the PCC and the dlPFC in the contrasts of high versus low violation severity, indicating the supportive function of the PCC in preventing norm breaches.

### Neural activation of punishment execution

Consistent with previous punishment research (e.g., Buckholtz et al.,^35^ Glass et al.,^33^ Stallen et al.^26^), we observed heightened activation in the left dlPFC when contrasting high versus low punishment conditions. Furthermore, higher levels of norm violations and psychological costs also induced increased dlPFC activity. This indicates that the left dlPFC serves as a common neural foundation, actively participating in the execution of social punishment. Notably, the dlPFC identified in our current paradigm is situated closer to the anterior region (BA 9) compared to the posterior part in previous studies (BA 44/46). ROI analyses further revealed that the posterior dlPFC (extracted from previous studies) failed to distinguish between high and low punishment conditions. These results underscore the role of the anterior dlPFC in social punishment.

Increased MTG activation has been linked to individuals’ willingness to punish during social interactions,^97^^,^^98^ as supported by meta-analysis evidence.^72^ Structural aspects of this region, such as gray matter volume, have been shown to positively predict an individual’s compliance with social norms.^99^ Consistent with previous research, ROI results revealed that the left MTG was specifically engaged in processing a strong propensity to punish (i.e., highly likely to stop). This is distinct from the dlPFC activation, which was observed across all levels of punishment tendency (both mildly and highly likely to stop). These findings suggest that the MTG is involved in the elaborative assessment of punishment execution in everyday social interactions where direct punishment is infrequent due to potential additional costs.

### Insights from a comprehensive brain network analysis

The brain is known for its efficient communication within networks, maintaining optimal function with minimal resource expenditure, a characteristic feature of small-world networks.^100^ Existing studies suggest that tasks with high difficulty or high cognitive load could increase the integration of the brain network, leading to higher global efficiency.^101^^,^^102^ The network analysis revealed that the current punishment network exhibits these small-world properties, indicating efficient transmission paths within the network. Furthermore, the global efficiency of the brain network was a strong predictor of individuals’ punishment tendencies, with a more significant effect observed in the high punishment tendency group. According to previous studies,^101^^,^^102^ the high demand for cognitive load of the current task may increase the integration of the brain network, leading to higher global efficiency. Therefore, individuals with high punishment tendency may process information about social norm violation more sufficiently. The tHOFC analysis demonstrated that the connections among the regions effectively distinguished between high and low punishment tendency individuals. In the high punishment tendency group, positive connections within brain regions processing violations were more pronounced, whereas negative connections between regions processing violations and psychological costs were stronger. The stronger negative connections between regions involved in processing violation severity and psychological costs might reflect a conflict processing between collective and personal interests, consistent with previous research showing inhibition between two neural networks (e.g., empathy processing^103^). Meanwhile, previous studies have found that people will sacrifice individual interests for collective interests social punishment is implemented.^19^^,^^104^ This finding suggests that neural activations related to collective interests could inhibit the neural processing of individual interests during social punishment decisions. This evidence suggests that individuals with high punishment tendencies have more streamlined brain activity, where the processing of social norm violations inhibits the consideration of psychological costs, thereby facilitating efficient altruistic punishment decision-making. These findings align with the decision tree analysis, which showed shorter psychological processing paths for high punishment tendencies. In conclusion, the findings suggest that individuals with a high punishment tendency may exhibit a stronger demand for social norm adherence and order, which results in greater integration and efficiency within the brain networks involved in processing social punishment. Regarding the topological characteristics of brain networks, indicators such as flexibility and resilience are of interest. Future research is encouraged to investigate how the characteristics of the social punishment network might differ from those involved in processing non-social punishment. For example, the flexibility of the social punishment network might be higher to adapt to various social norms and interactions in the contexts.

Previous researchers have proposed a neural punishment network based on economic and criminal scenarios. This network encompasses the salience network (e.g., dACC, AI) for detecting violations, the default mode network (e.g., vmPFC) for evaluating injury and violator’s intention, and the central execution network (e.g., dlPFC) for making punishment decisions.^1^^,^^30^ The efficacy of this punishment network in fairness norms and its predictive ability for individual punishment tendencies in economic paradigms has been widely confirmed.^105^ The current study complements this line of research. First, it provides insights into the neural substrates of norm-related processing during punishment decisions. Instead of evaluating economic metrics like unfairness or loss, the severity of norm violations is appraised based on an inherent aversion to behaviors that undermine cooperation.^106^ Contrary to earlier studies that have identified the anterior insula and anterior cingulate cortex (especially the dorsal ACC) as central to processing value-based unfairness in economic punishment,^1^^,^^25^^,^^26^^,^^30^^,^^107^ our current findings highlight the involvement of the fusiform gyrus and the PCC, suggesting that punishment within social contexts is driven by both emotional (social aversion) and cognitive (prevention-focused) mechanisms. The activation of the fusiform gyrus suggests the neural basis for aversion to norm violations^108^^,^^109^ and threatening outgroups.^110^ Supporting this notion, research on infants and young children shows that such social aversion is evident as early as 6 months of age, whereas the ability to detect value-based violations does not appear until around 3 years of age.^9^^,^^111^ These findings reinforce the idea that punishment for norm violations is altruistic in nature.

To sum up, there are both overlapping and unique functions between the current punishment network and the punishment-related regions activated in previous research. First, the current punishment network shares an overlap with previous punishment networks, particularly in the dlPFC, which was involved in the execution of punishment decisions. However, although this region (especially the anterior part) forms a common foundation, social punishment also recruits the MTG to support the detailed assessment and elaboration of punishment execution. Second, social punishment uniquely engages regions such as the fusiform gyrus and PCC, suggesting a blend of emotional and cognitive responses specifically tied to social norm violations. Third, the cognitive functions of the vmPFC and MCC regions activated in the current paradigm and previous punishment research seem different. While the activation of the two regions is involved in the assessment of harm experienced by victims in previous research,^1^^,^^30^ the current findings suggest that the two regions are engaged in evaluating the extent of psychological costs in the social context. These findings emphasize the overlapping and unique cognitive and neural functions of social punishment network when compared to the punishment-related activations and circuits established in previous economic and criminal paradigms.

This study highlights the collaborative dynamics among subnetworks within the punishment brain network. Behavioral findings indicate that participants prioritize the severity of norm violations over the assessment of psychological costs. The analysis of the punishment network suggests that processing the severity of violations seems to interfere with the assessment of psychological costs, highlighting a complex interplay between different cognitive processes involved in punishment decision-making. Our research identifies overlaps between the new neural network of social punishment and the established punishment network, particularly in regions associated with harm evaluation (i.e., the default mode network, DMN) and punishment implementation (i.e., the central executive network CEN). Previous studies have shown that resting-state functional connectivity, particularly within the frontal-parietal network (FPN), can predict punishment tendencies.^34^ In consistent with this, the resting-state connectivity within the established network can distinguish between individuals with high and low punishment tendencies, albeit with lower classification accuracy.

### Conclusion and future directions

Our findings introduce a new framework for social punishment, involving two cognitive pathways: “the *C* (collective) path,” focusing on the severity of norm violations for collective interests, and “the *P* (personal) path,” centered on the assessment of psychological costs for personal interest protection (Figure 3D). The decision tree results suggest that individuals tend to prioritize the *C* path over the *P* path when administering punishment in social contexts. More severe violations activate the *C* path, bypassing the *P* path. At the neural level, higher levels of violation severity heighten internal functional connectivity within regions responsible for processing violation severity, such as the fusiform gyrus and PCC, which triggers the *C* path for social punishment and results in a high punishment tendency. Conversely, punishment decisions for lower levels of violation severity predominantly rely on the *P* path, engaging brain regions associated with processing psychological costs, such as the MCC and vmPFC regions. This can lead to either high or low punishment tendencies, depending on psychological costs. Elevated psychological costs drive a propensity for heightened punishment, whereas diminished psychological costs correspond to a reduced punishment tendency. Further analysis of RTs underscores the contribution of the *C* path to enhancing the efficacy of social punishment. Brain network analysis highlights that heightened activation of brain regions associated with the *C* path is linked to a corresponding reduction in activation intensity of the *P* path.

### Limitations of the study

This study opens new avenues for future research. While the current paradigm focuses on static queues, social punishment in dynamic environments also deserves attention. Future studies should examine social punishment in dynamic contexts using methods such as field studies. While the current paradigm maintains a balance of conditions, certain scenarios (e.g., a queue-jumper cutting in at the back of the queue) may occur less frequently than the other ones in real life (see the supplemental information on relative position for details). We suggest that future research investigates how the relative position at which a queue-jumper cuts in line may influence individuals’ perception of the jumper’s intention, potentially affecting subsequent punishment decisions. Preliminary analyses showed the relative position between queue-jumpers and individuals could influence the propensity for punishment (see the supplemental information on relative position and Table S16 for details). Future rekey search is encouraged to explore how interpersonal factors, such as physical and psychological distance, would modulate the cognitive and neural mechanisms underlying social punishment. We suggest future research to consider different social violation scenarios and design other new paradigms simulating scenarios that would be encountered by people in daily situations. Additionally, altruistic behavior and norm enforcement can vary across cultures. Consequently, it remains to be seen whether the findings of this study can be universally applied to other cultures. Future studies are encouraged to examine how cultural differences influence individuals’ perceptions of social norms and their tendencies toward punishment. Lastly, research should explore novel interventions aimed at fostering prosocial behavior, which could include promoting norm sensitivity, empathy-building exercises, and other strategies designed to reduce antisocial behavior and enhance cooperation in social groups. Regarding the specific mechanisms identified in this study for the influence of neural activity on collective and personal interests, we encourage future studies to employ novel methodology for further exploration. Additionally, we advocate for future research to utilize advanced methodology, including computational modeling, to further determine the computational mechanisms underlying processing collective and personal interests within the realm of social punishment in real-life contexts.

## Resource availability

### Lead contact

Further information and requests for resources should be directed to the lead contact, Yan Mu (muy@psych.ac.cn).

### Materials availability

This study did not generate new unique reagents.

### Data and code availability

• Data: the fMRI and behavior data used to generate the results in this study have been deposited in the Science DataBank dataset and are publicly available as of the date of publication. DOIs are listed in the key resources table.
• Code: the original code has been deposited in the Science DataBank dataset and is publicly available as of the date of publication. DOIs are listed in the key resources table.
• Any additional information required to reanalyze the data reported in this paper is available from the lead contact upon request.

## Acknowledgments

This work was supported by the National Natural Science Foundation of China (32071016, 32271129, 32471126), and CAS Key Laboratory of Behavioral Science, Institute of Psychology, Chinese Academy of Sciences (2019000050). We thank Hanchun Yang, Chunan Yao, Tianque Gao, Xinni Wang, Jianing Zhang, and Zhiyan Yang for assisting with data collection and Yunhan Liu for helping with stimuli generation. We thank Zhiruo Ni for helping us to check the experimental materials used in the Supplementary Study 4.

## Author contributions

Conceptualization, Y.M. and J.Z.; methodology, J.Z. and X.L.; investigation, J.Z. and X.Z.; writing—original draft, J.Z., X.Z., X.L., and Y.M.; writing—review & editing, J.Z., X.Z., X.L., and Y.M.; funding acquisition, Y.M.; supervision, Y.M.

## Declaration of interests

The authors declare that they have no competing interests.

## STAR★Methods

### Key resources table

**Table:** 

REAGENT or RESOURCE	SOURCE	IDENTIFIER
**Deposited data**		

fMRI and behavior data	This study	Science Data Bank: https://doi.org/10.57760/sciencedb.16788
Original code	This study	Science Data Bank: https://doi.org/10.57760/sciencedb.16788

**Software and algorithms**		

Psychopy	Peirce	https://www.psychopy.org
Pavlovia	PsychoPy Team	https://pavlovia.org
Jamovi	The jamovi project	https://www.jamovi.org/about.html
STATA (version 17)	StataCorp LLC	https://www.stata.com/stata17
R (version 4.2.2)	The R Project for Statistical Computing	https://www.r-project.org
rpart	Therneau et al.^63^	https://cran.r-project.org/package=rpart
brms	Bürkner^76^	https://rdrr.io/cran/brms
caret	Kuhn^112^	https://CRAN.R-project.org/package=caret
lme4	Bates et al.^113^	https://cran.r-project.org/package=lme4
Matlab_R2019b	Math Works, Natick, MA, USA	https://it.mathworks.com/products/matlab.html
Statistical Parametric Mapping (SPM 12)	Wellcome Department of Imaging Neuroscience,London, UK	https://www.fil.ion.ucl.ac.uk/spm/software/download/
Decoding Toolbox	Hebart et al.^66^	https://sites.google.com/site/tdtdecodingtoolbox
PPPI V3	NITRC (Neuroimaging Tools and Research Collaboratory)	https://www.nitrc.org/projects/ppi_batch_hipp
DPABI (V5.1_201201)	Resting-State fMRI Data Processing Assistant (DPABI)^114^	https://rfmri.org/DPABI
BrianNetClass	Zhou et al.^69^	https://github.com/zzstefan/BrainNetClass

### Experimental model and study participant details

#### Human participants

##### fMRI study

To ensure adequate statistical power, we recruited a total of 60 right-handed, healthy adults with no history of psychiatric or neurological disorders through advertisements in Beijing. This sample aligns with recent fMRI studies on punishment, where participant numbers ranged from 20 to 55 (e.g., N = 20^25^; N = 28^115^ ; N = 55.^105^ One participant was unable to complete the experiment due to discomfort in the scanner, resulting in 59 valid datasets (27 males, mean ± SD age = 24.22 ± 4.69 years). Prior to participation, all participants were briefed on the research objectives and provided informed consent. Each participant received 150 RMB for involvement.

##### Supplementary Study 1

In the preregistration (https://aspredicted.org/N4D_693), we planned to enroll 250 participants. During the online recruitment, several participants completed the study almost at the same time. Thus, we ended up with 255 samples. First, we excluded testing data from 2 laboratory assistants who helped with checking the experiment link. Second, as stated in the preregistration, we excluded 6 participants that answered the attention check questions incorrectly. Finally, the final sample size was 247 (115 males and 132 females, mean age = 22.08 ± 3.29 years). Each participant received 15RMB as compensation.

##### Supplementary Study 2

We pre-registered this study (https://aspredicted.org/vkmy-jkgs.pdf) and recruited 200 participants. After excluding six participants that answered the attention check questions incorrectly, the final sample size was 194 (100 males and 194 females, mean age = 22.40 ± 3.38 years). A compensation of 15 RMB was provided to each participant.

##### Supplementary Study 3

We pre-registered this study (https://aspredicted.org/wf7m-j5vg.pdf) and recruited 200 participants, including 94 law students and 106 non-law students. In law group, we excluded 8 participants that answered the attention check questions incorrectly. The final sample size of the law group was 86 (35 males and 51 females, mean age = 22.35 ± 3.22 years). There were four participants were excluded for answering the attention check questions incorrectly. The final sample size of the non-law group was 102 (48 males and 54 females, mean age = 21.35 ± 3.01 years). Each participant received 15 RMB as compensation.

##### Supplementary Study 4

We recruited 32 participants (14 males and 17 females, one participant was reluctant to disclose the gender and age information; mean age = 21.48 ± 1.71 years). Each participant was given 40 RMB as remuneration.

The experiments involving human participants in this research have been reviewed and approved by the Ethics Committee of the Institute of Psychology, Chinese Academy of Sciences. The participants provided their written informed consent to participate in this study.

### Method details

#### fMRI study

##### The queue-jumping paradigm

Fifty-nine participants were instructed to imagine themselves queuing with five others to purchase movie tickets (Figure 1A). We manipulated six distinct positions for the queue-jumper to represent varying levels of norm violation severity; the closer the queue-jumper was to the front of the queue, the more severe the norm violation. Additionally, we varied participants’ positions in the queue to manipulate six levels of personal psychological costs; proximity to the end of the queue resulted in greater psychological costs due to the anticipated increase in waiting time. To examine the impact of resource scarcity on punishment decision-making, we manipulated two levels of remaining tickets (sufficient or insufficient). Consequently, this created thirty-six stimuli (6×6) for both sufficient and insufficient ticket conditions. Out of these thirty-six stimuli, twenty-one involved self-involvement, where the queue-jumper was positioned in front of and impacted the participant. The remaining fifteen stimuli, where the queue-jumper was positioned behind and did not affect the participant, involved minimal self-involvement. To balance the duration of self-involvement and non-involvement trials and minimize interference with brain activity, one “involvement” stimulus and seven “non-involvement” stimuli were randomly selected from both sufficient and insufficient conditions before each experiment. These stimuli were presented twice. Subsequently, eighty-eight trials were randomly assigned to two experimental runs. During each trial, the queue-jumping stimulus was displayed for 1.5 seconds, after which participants rated their subjective punishment tendency on a four-point scale (with higher scores indicating a greater intention to punish) within the next 3.5 seconds. This rating was followed by a jittered fixation period (seven durations ranging from 0.5s to 7.5s) averaging 3 seconds. We evaluated participants’ willingness to intervene and prevent a queue-jumper using a 4-point scale (1 = highly unlikely to stop, 4 = highly likely to stop). For a detailed assessment of the ecological validity using a four-point scale to gauge participants’ punishment tendency, please refer to the “ecological validity and generalizability of the queue-jumping paradigm” section in the main text and the STAR Methods section. The experiments were programmed using PsychoPy, an open-source software package primarily designed for neuroscience and experimental psychology research, written in Python.^116^

##### Neuroimaging acquisition

MRI data were obtained using a 3.0 Tesla General Electric MR system (GE 3.0 T scanner: Discovery MR750) at the Magnetic Resonance Imaging Research Center of the Institute of Psychology, Chinese Academy of Sciences. Task-dependent fMRI data were obtained using a T2-weighted echo-planar imaging (EPI) protocol with the following parameters: repetition time (TR) = 2000 ms, echo time (TE) = 30 ms, 37 axial slices with a slice thickness of 3.5 mm, flip angle = 90°, field of view (FOV) = 64 × 64 mm^2^, and interleaved slice ordering. There were three task runs, each consisting of 185 volumes. T1-weighted anatomical images were acquired with the following parameters: TR = 6.7 ms, TE = 2.9 ms, 192 slices, flip angle = 12°, FOV = 256 × 256 mm^2^, and in-plane resolution = 1 × 1 mm^2^. A total of 192 volumes were acquired using a Magnetized 3D rapid gradient-echo pulse sequence. Following T1-weighted scanning, resting-state scanning was performed for 8 minutes, yielding 240 volumes. During the resting-state scanning, participants were instructed to relax and keep their eyes closed. The scanning parameters were consistent with those used in the task runs.

#### Supplementary Study 1

This study aimed to assess the ecological validity of the queue-jumping paradigm by exploring whether participants’ tendency to punish in this context correlates with the frequency of their social punishment behaviors in everyday life.

##### Measures and paradigms

We measured a variety of punishment tendencies, including queue-jumping punishment, economic punishment, criminal punishment, social norm punishment, and the frequency of participants’ interventions to prevent common social norm violations in their everyday lives. Lastly, we collected participants’ demographic variables (e.g., age and gender). See below for details.

(a) **Queue-Jumping Punishment.** To confirm the impacts of violation severity and psychological costs, we employed a similar set of 36 queue-jumping scenarios (6 levels of violation severity x 6 levels of psychological costs) as those used in the fMRI study, with the exception that we did not manipulate resource scarcity and the stimuli were presented once. To keep consistent with other paradigms, we modified the original 4-point scale and assessed participants’ punishment tendencies on a 7-point scale (1= very unlikely to punish to 7 = very likely to punish).

Next, participants were asked to rank a set of representative scenarios based on violation severity and psychological costs. We used a simplified set of representative stimuli involved three positions (rather than the six used in the fMRI study) for participants and queue jumpers: the first, third, and sixth rows. In total, there were nine queue-jumping scenarios. The instruction for ranking violation severity was: “Please rank the degree of violation in the following scenarios from high to low”. The guidance for ranking psychological costs ranking was: “Please rank the degree of psychological costs caused by queue jumping in the following scenarios”.

Finally, we assessed participants’ motivation to stop queue jumpers to understand the behind psychological processes when making punishment decisions. Specifically, participants were asked to rate the extent to which each of the following items influenced their motivation to intervene on a 7-point scale (from 1 = strongly disagree to 7 = strongly agree): (1) queue jumpers violate social norms, (2) queue jumping behaviors increase their waiting time, (3) queue jumping behaviors affect their ticket purchase, (4) queue jumping behaviors impact the interests of others in the queue, (5) queue jumping behaviors cause chaos, and (6) queue jumping behaviors violate personal norms. Additionally, we asked participants to rank the following items related to psychological costs in term of their perceived impact: increasing my waiting time, increasing risks of missing out on the opportunity to buy tickets, and violating the social norms that I follow. The highest-ranked cost was considered the main psychological cost perceived by participants.

(b) **Economic Punishment.** Consistent with previous economic punishment research,^26^ we informed participants that they were playing a three-person game and had been assigned the role of Player A based on their date of birth (in reality, all participants were assigned Player A). Participants were told that their partners (Player B and Player C) had participated in a reaction speed experiment and the two players performed equally well in the last round. Player B won a 6-yuan bonus through a lottery and could decide how to distribute this bonus between themselves and Player C. Player C had no choice but to accept the distribution. Participants assigned the role of Player A acted as a judge with 3 yuan. They could either reduce Player B’s income by punishing Player B or increase Player C’s income by donating to Player C. We informed them there were two options: 1) the first option with a loss ratio of 1:3, meaning that for every 1 yuan the participant spent to punish Player B, Player B’s income was reduced by 3 yuan; the second option with a gain ratio of 1:3, meaning that for every 1 yuan the participant spent to donate to Player C, Player C’s income increased by 3 yuan. Participants’ punishment for player B was considered social punishment, regardless of the amount spent.
(c) **Criminal Punishment.** According to previous research on criminal punishment,^24^ we asked to participants to rate their punishment tendencies in eight criminal situations on a 7-point scale (1 = should not be punished at all, 7 = should be punished severely). These situations included four intentional violations (e.g., intentional homicide; Cronbach’s alpha coefficient = 0.62) and four unintentional violations (e.g., unintentional homicide; Cronbach’s alpha coefficient = 0.83). Participants’ tendencies to punish for intentional and unintentional crimes were averaged separately to indicate their propensity for punishment in the criminal contexts.
(d) **Social Norm Punishment.** According to previous research on punishment for a variety of social norm violations,^22^ we asked participants to rate their punishment tendencies in a set of social norm violation stimuli on a 7-point scale (1 = should not be punished at all, 7 = should be punished severely): five intentional violations (e.g., intentionally spilling a Coke on a friend; Cronbach’s alpha coefficient = 0.66), five unintentional violations (e.g., accidentally spilling a Coke on a friend; Cronbach’s alpha coefficient = 0.77), and five neutral behaviors (e.g., sharing a Coke with a friend; Cronbach’s alpha coefficient = 0.61).We averaged participants’ scores in each of the three conditions, with higher scores indicating with higher scores indicating greater tendencies toward punishment for norm violations.
(e) **Punishment frequency in daily life.** Adapted from previous research on social norm violations,^79^ we measured the frequency of punishment in daily life by assessing participants’ responses to ten scenarios involving typical social norm violations. Specifically, we examined how often participants engage in preventing violations of social norms in everyday situations on a 7-point scale (1 = never, 7 = always), such as making loud phone calls or playing music in public places. We calculated the average frequency of punishment across all scenarios to index participants’ punishment practices in everyday life. A higher average value indicates a greater frequency of engaging in social punishment. The Cronbach’s alpha coefficient for this scale was 0.90.

#### Supplementary Study 2

This study aimed to future evaluate the ecological validity and generalizability of the queue-jumping paradigm. Specifically, we tested whether the queue-jumping paradigm effectively evokes a real-world queue-jumping scenario, and examined whether participants’ punishment tendencies in the paradigm predict their actual social punishment practices in real-life situations.

##### Measures and paradigms

Firstly, consistent with Supplementary Study 1, we measured a variety of punishment tendencies, including queue-jumping punishment, economic punishment, criminal punishment, social norm punishment, and the frequency of participants’ interventions to prevent common social norm violations in their everyday lives. Secondly, to enhance the ecological validity of the queue-jumping paradigm as well as the generalizability of the queue-jumping, we further measured participants’ actual punishment practices in their daily lives. These measures facilitate our examination of whether participants’ punishment tendencies within the queue-jumping paradigm are predictive of their social punishment behaviors in real-life scenarios. Lastly, we collected participants’ demographic variables (e.g., age and gender). Further details are provided below.

(a) **Queue-Jumping Punishment.** The queue-jumping punishment task was consistent with Supplementary Study 1. After the queue-jumping punishment task, we assessed participants’ motivation to stop queue jumpers to understand the behind psychological processes when making punishment decisions. The measurement was in line with Supplementary Study 1. Additionally, we measured the perceived costs associated with participants’ willingness to intervene in queue-jumping incidents. Participants were required to choose from four predefined options the potential looses they might face as a consequence of preventing queue-jumping, based on their everyday life experiences. The four potential looses include: demage their public image, time loss, causes financial losses, potential interpersonal conflicts. Finally, to test whether the queue-jumping paradigm effectively evokes a real-world queue-jumping scenario, we assessed how easily the paradigm evoked participants’ recollections of real-life queue-jumping scenarios on a seven-point Likert scale (1 = *very difficult* to 7 = *very easy*).
(b) **Economic Punishment.** This measure was consistent with Supplementary Study 1.
(c) **Criminal Punishment.** This measure was consistent with Supplementary Study 1. Cronbach’s alpha coefficient for the four intentional violations was 0.61, while for the four unintentional violations, it was 0.82.
(d) **Social Norm Punishment.** This measure was consistent with Supplementary Study 1. Cronbach’s alpha coefficient was 0.68 for the five intentional violations, 0.79 for the five unintentional violations, and 0.40 for the neutral behaviors.
(e) **Punishment frequency in daily life.** This measure was consistent with Supplementary Study 1. Cronbach’s alpha coefficient was 0.88.
(f) **Actual punishment practice in daily life.** To obtain participants’ actual punishment practices in everyday situations, we referred to a study with high ecological validity that employed a diary method to measure participants’ punishment behaviors within their personal experiences of social norm violations.^12^ The assessment of participants’ everyday punishment practices consists of two sections.

In the first section, participants were asked to recall a real-life queue-jumping scenario they had encountered, including details such as: the time and place of the incident, the purpose of the queue, the approximate number of people in line, a description of the queue-jumping scenario, the position of the queue-jumper (at the front, middle, or back of the line), their distance from the queue-jumper (whether the queue-jumper was close to them), and the relative position of the queue-jumper (in front of or behind them). Subsequently, participants were presented with seven types of punishment strategy (see numbers identified as follows) and asked to rate their willingness to implement each punishment strategy at that moment on a seven-point scale (1 = not at all, 7 = very much), with higher scores signifying a stronger inclination to employ the respective strategy. Then, participants used a two-point scale (yes or no) on each of the seven strategies to recall whether they had adopted the strategy. Drawing from the research of Molho et al.,^12^ the seven types of punishment behaviors encompass both direct and indirect punishment. Direct punishment behaviors include taking action to deter queue-jumping ***(1)*** and verbally stopping the queue-jumper ***(2)***, while indirect punishments involve sharing negative information about the queue-jumper with others (i.e., gossip) ***(3)*** and excluding them from future social interactions ***(4)***. To determine the extent and preferences of costs participants are willing to bear in punishing queue-jumping behavior, we introduced two additional indirect punishment items: one addressing the time cost of making a complaint against the queue-jumper ***(5)*** and another concerning the financial cost of preventing such behavior ***(6)***. Additionally, we introduced an item for “unwillingness to take any action ***(7)***,” which, when reverse-scored, indicates participants’ willingness to take measures to prevent queue-jumping behavior.

At the beginning of the second part, participants first read the definition of social norm violation: *“Social norm violations” generally refer to actions that breach unwritten but widely observed social norms during social activities, which often impact others’ social engagements.* Participants were then asked to recall a recent social norm violation incident they had experienced or witnessed. Similar to the first part, participants rated their willingness to implement each punishment strategy on a seven-point scale (1 = not at all, 7 = very much) and used a two-point scale (yes or no) on each of the seven strategies to recall whether they had adopted the strategy. Unlike the first part, all descriptions related to “queue-jumpers” were replaced with “social norm violators.” Additionally, participants were required to recall details about the social norm violation incident they reported, including: their social relationships with the violators (relatives and friends, classmate/colleague, stranger), the number of people present during the incident, the perceived intent of the violator (1 = *absolutely not intentional*, 7 = *completely intentional*), the impacts of the social norm violation on the participant and others present (two separate items; 1 = *no impact*, 7 = *extremely high impact*), and participants’ positive and negative emotions experienced (two separate items; 1 = *very few positive/negative emotions*, 7 = *a lot of positive/negative emotions*).

#### Supplementary Study 3

Building on Supplementary Study 2, we further investigate the generalizability of the queue-jumping paradigm by examining whether participants’ punishment tendencies within this paradigm vary based on their professional backgrounds and experiences. Specifically, whether individuals from law backgrounds exhibit a higher propensity for punishment than those form non-law backgrounds.

##### Measures and paradigms

First, consistent with Supplementary Studies 1 and 2, we measured a variety of punishment tendencies, including queue-jumping punishment, economic punishment, criminal punishment, social norm punishment, the frequency of participants’ interventions to prevent common social norm violations and their actual punishment practices in their daily lives. To validate the professional training of the law group, we used five representative multiple-choice questions related to law (e.g., “The Difference Between Case Law and Statutory Law”), which were checked and reviewed by a PhD student in law. As predicted, the law group exhibited a higher accuracy in these questions than the control group. The accuracy of the two groups was 74.65% (law) and 50.39% (non-law), respectively; *t* = 7.72, *p* < 0.001. Lastly, we collected participants’ demographic variables (e.g., age, gender, and major). Further details are provided below.

(a) **Queue-Jumping Punishment.** The measure was consistent with Supplementary Study 2.
(b) **Economic Punishment.** The measure was consistent with Supplementary Studies 1 and 2.
(c) **Criminal Punishment.** The measure was consistent with Supplementary Studies 1 and 2. Cronbach’s alpha coefficients for the four intentional violations were 0.32 and 0.51 for the law group and the non-law group, respectively. Cronbach’s alpha coefficients for the four unintentional violations were 0.86 and 0.85 for the law group and the non-law group, respectively.
(d) **Social Norm Punishment.** The measure was consistent with Supplementary Studies 1 and 2. Cronbach’s alpha coefficients for the five intentional violations were 0.71 and 0.76 for the law group and the non-law group, respectively. Cronbach’s alpha coefficients for the five unintentional violations were 0.67 and 0.66 for the law group and the non-law group, respectively. Cronbach’s alpha coefficients for the five natural behaviors were 0.59 and 0.71 for the law group and the non-law group, respectively.
(e) **Punishment frequency in daily life.** The measure was consistent with Supplementary Studies 1 and 2. Cronbach’s alpha coefficients were 0.90 for the law group and 0.91 for the non-law group.
(f) **Actual punishment practice in daily life.** The measure was consistent with Supplementary Study 2.

#### Supplementary Study 4

Given that real-life punishment is a binary choice, i.e., punishing or not. We aimed to test the relationship between the four-point punishment tendency rating scale and the binary punishment decision. We then conducted an experiment employing a within-subjects design to explore the correlation between the two types of punishment measures.

##### Measures and paradigms

The experiment was conducted online using the Pavlovia platform (https://pavlovia.org). The design mirrored that of the fMRI experiment, with the primary distinction lying in the response method. Participants were required to rate their punishment tendency on a four-point scale, aligning with the parameters of the fMRI study (1 = highly unlikely to intervene, 4 = highly likely to intervene) in one experiment session, and make a binary decision to either stop the queue-jumper or not in another session. The study employed a within-subjects design, with participants attending two experimental sessions spaced two days apart. To control for order effects, the sequence of the two sessions was counterbalanced between participants.

### Quantification and statistical analysis

#### Behavioral data analyses

First, we employed Analysis of Variance (ANOVA) to assess whether there were significant differences in participants’ inclination to administer social punishment across various experimental conditions, thereby validating the effectiveness of our manipulations. Two repeated measures ANOVAs (one for violation severity and another for psychological costs) and a paired sample t-test (for resource scarcity) were conducted using Jamovi (https://www.jamovi.org/about.html). Subsequently, a series of regression analyses were performed to examine how participants’ propensity to administer punishment varied based on violation severity, psychological costs, and resource scarcity. Considering the differences in levels of the three factors, we centralized and standardized the three key independent variables before regression analyses. An ordered probit regression analysis, which is particularly suited for ordered data,^75^ was conducted using STATA 17 (https://www.stata.com/stata17) to investigate the presence and pattern of our manipulations’ impact on participants’ punishment tendencies, specifically the directional influence. The analysis indicated that violation severity had the most substantial marginal effect on participants’ inclination to administer punishment, followed by psychological costs among the three specified variables. Additionally, a decision tree analysis was employed to delineate participants’ psychological processing pathways in social punishment using the R package *rpart*.^63^ Lastly, a cumulative ordered logistic regression was performed to further explore the relationship between punishment propensity and reaction time using the R package *brms*.^76^ The Bayesian model was specified using default priors. For the intercept and random effects, weakly informative priors based on Student’ s t distributions (3, 0, 2.5) were applied to enhance model stability. Flat (non-informative) priors were used for all slope coefficients to ensure that the analysis remained data-driven and unbiased by prior beliefs. See details on these analyses in the supplemental information for Punishment Tendency and Reaction Time.

#### Neuroimaging data analyses

The fMRI data were preprocessed using Data Processing and Analysis for Brain Imaging (DPABI^114^). First, univariate and multivariate analyses were conducted for identifying regions that show significant activation differences between conditions and searching for unique neural patterns distinguishing between different condition levels that might have been overlooked by univariate analysis. We used univariate analysis to examine the differences in average brain activation intensity when contrasting different levels of violation severity, psychological cost, and resource scarcity conditions, as well as to examine the brain activation intensity of varying levels of punishment tendencies with SPM12 (http://www.fil.ion.ucl.ac.uk/spm). Brain regions reaching cluster-level significance at *p* < 0.05 (FWE correction) were reported, with a cluster-forming threshold of *p* < 0.001^117^ and a cluster size k ≥ 30 voxels. A small volume correction (8 mm spheres) was applied with a threshold of *p* < 0.05 (FWE correction) to prevent missing potentially significant activations due to stringent thresholds. Second, we conducted multivariate analyses to further explain the brain activation patterns in the aforementioned contrasts. A linear support vector machine (SVM) algorithm was utilized and implemented via the Decoding Toolbox.^66^ Group-level statistical analyses were performed using one-sample t-tests to assess the significance of the decoding accuracy across participants. We defined the brain regions identified by univariate and multivariate analyses as regions of interest (ROI). Generalized Psychophysiological Interaction (gPPI) analyses were conducted using the PPPI toolbox^67^ to identify the task-related regions associated with specific conditions, search for unique neural patterns distinguishing between different condition levels and to pinpoint the connected brain areas potentially interacting with punishment-related regions. The significance threshold for the gPPI results was set at p < 0.05 and FWE corrected, with a cluster-forming threshold of p < 0.005 (a commonly used threshold in gPPI analysis^118^^,^^119^ and a cluster size ≥ 30 voxels. To depict a global neural architecture for social punishment, brain network analysis was used to reveal the functional coupling of brain regions over time. We generated the tHOFC networks using the BrainNetClass toolbox.^69^ LASSO regression was used to remove links with low contribution values from the model.^69^ Refer to the details below.

#### Preprocessing

The fMRI data were preprocessed using Data Processing and Analysis for Brain Imaging (DPABI^114^) with the following steps. The original DICOM data were converted to the NIFTI format. Subsequently, temporal and spatial corrections were performed on the data. Head motion thresholds were set to ensure a maximum translation of less than 3mm and a maximum rotation of less than 3 degrees. All subjects included in the study met these criteria. The 179 images per participant were registered and normalized to the standard space of the Montreal Neurological Institute (MNI) using the EPI template (voxel size = 3mm×3mm×3mm). The Friston 24-parameter model was utilized to regress out head motion effects from the realigned data. Finally, the functional data were spatially smoothed with an 8mm full-width-at-half-maximum (FWHM) Gaussian kernel.

#### Univariate analysis

The preprocessed MRI images underwent further analysis using general linear models (GLM) with SPM12 (http://www.fil.ion.ucl.ac.uk/spm). The six head movement correction parameters were included as regressors in the model. Each regressor was convolved with a canonical hemodynamic response function (HRF), considering both temporal and dispersion derivatives. Subsequently, the resulting statistical maps were then subjected to second-level random-effect analyses to identify group-level activations. Brain regions reaching cluster-level significance at *p* < 0.05 (FWE correction) were reported, with a cluster-forming threshold of *p* = 0.001 and a cluster size k ≥ 30 voxels. A small volume correction (8 mm spheres) was additionally applied with a threshold of *p* < 0.05 (FWE correction) to prevent missing potentially significant activations due to stringent thresholds.

#### Multivariate analysis

To examine participants’ neural patterns in response to varying levels of violation severity and psychological cost conditions, we employed a multivariate pattern analysis (MVPA) utilizing a 5mm-radius region of interest (ROI) whole-brain searchlight approach. A linear support vector machine (SVM) algorithm was utilized and implemented via the Decoding Toolbox.^66^ The SVM was trained to differentiate between conditions of high and low levels of violation severity, as well as high and low psychological costs. Subsequently, voxel-wise decoding accuracy maps were created based on the searchlight analysis. The accuracy maps were normalized to Montreal Neurological Institute (MNI) space and then smoothed with an isotropic Gaussian kernel with a full width at half maximum (FWHM) at 8 mm. Group-level statistical analyses were performed using one-sample t-tests to assess the significance of the decoding accuracy across participants.

#### ROI analysis

The signals of ROIs were extracted using the DPABI toolbox^114^ and were centered at the coordinates of the significantly activated brain regions from the aforementioned GLM analyses. To compare activation across various conditions (e.g., high versus low violation severity, high versus low psychological costs, and resource insufficiency versus sufficiency) (refer to the Supplementary Results on neural results of resource scarcity), we selected ROIs that showed significant activation in the respective contrasts. For example, the fusiform gyrus (FG) was used for processing violation severity; the ventromedial prefrontal cortex (vmPFC) for processing psychological costs; the temporoparietal junction (TPJ) for processing resource scarcity, as detailed in the Supplementary Results on neural outcomes of resource scarcity. Paired sample t-tests were conducted to examine differences in ROI activation across different conditions. In the generalized psychophysiological interactive (gPPI) analysis, neural activation from different contrasts served as the sources for ROI seeds to evaluate gPPI connections. For instance, brain regions significantly activated in the contrast between high and low violation conditions (e.g., dlPFC) were used as seeds to further explore gPPI functional connectivity in the same contrast. Specific details regarding the gPPI seeds can be found in Table S8.

#### gPPI analysis

Generalized Psychophysiological Interaction (gPPI) analyses were conducted using the PPI toolbox^67^ to explore functional connectivity in the brain during punishment decision-making across different conditions. gPPI seeds were created by placing a 10-mm-radius sphere around the local maxima of the ROIs identified in the GLM analyses (see Table S8 for details). Given that the gPPI analysis is based on the convolution of the canonical hemodynamic response function (HRF), the first-level analysis was re-performed without incorporating the temporal and dispersion derivatives. Subsequently, the resulting SPM images from this adjusted first-level analysis were then used in the gPPI analysis. Group-level analysis for the gPPI mirrored the procedures in the GLM analyses. The significance threshold for the gPPI results was set at *p* < 0.05 and FWE corrected, with a cluster-forming threshold of *p* < 0.005 and a cluster size ≥ 30 voxels.

#### Brain network analysis

To depict a global neural architecture for social punishment, brain network analysis was used to reveal the functional coupling of brain regions over time. Considering that the decision-making of punishment involves three psychological processes related to violation severity, psychological costs, and punishment execution (i.e., the core node of the decision tree), we selected 19 brain regions processing the above three psychological processes as the nodes of the brain network construction based on the results of GLM, MVPA, and gPPI analysis. Among them, there are 9 nodes of processing violation severity (e.g., fusiform and PCC), 8 nodes of processing psychological costs (e.g., MCC and vmPFC), and 2 nodes of processing punishment execution (i.e., dlPFC and MTG), see Table S9 for details of coordinates of the ROIs composing the brain network. We then generated the tHOFC networks using the BrainNetClass toolbox.^69^ This approach initially assessed the similarity of topological profiles among ROIs. It involved calculating the first-order feature by taking the Pearson-based functional connectivity (FC) between the time series of each ROI and all other ROIs. Subsequently, the Pearson correlation matrix of the first-order features for each ROI was computed. Given the brain parcellation atlas with N ROIs, the fMRI signal at the ith ROI can be represented as a column vector xi =[x1i, x2i, ..., xTi]’ ∈ RT (’denotes transpose operation), and a data matrix X =[x1, x2, ..., xN] ∈ RT × N. Pearson correlation-based brain functional network can be represented as a graph with an edge weight matrix W ∈ RN × N whose element w is calculated by pairwise temporal correlation of the raw BOLD signals. The formula of tHOFC is as follows (where k is the number of clusters (new nodes) for clustering dynamic FC time series, wi· ={w ik|jk ∈ N, k≠i} and i, j, k =1,2,..., N, k ≠ i, j):

$${\text{tHOFC}}_{ij}=\frac{\sum_{k}\left(w_{ik}-{\bar{w}}_{i\cdot }\right)\left(w_{jk}-{\bar{w}}_{j\cdot }\right)}{\sqrt{{\sum_{k}\left(w_{ik}-{\bar{w}}_{i\cdot }\right)}^{2}}\sqrt{{\sum_{k}\left(w_{jk}-{\bar{w}}_{j\cdot }\right)}^{2}}}$$

To explore the similarities and differences between our brain network and the mature brain network on punishment processing proposed by predecessors, we analyzed the existing brain network related to punishment by tHOFC. The previously proposed punishment-related brain networks, which include the salience (SN), default mode (DMN), and central executive networks (CEN),^1^ have been fully validated both in functional connectivity and predictive capability for economic punishment.^105^ In the current study, the masks of the three brain networks were derived from the Power 264 template.^120^ Additionally, we employed sparse representation (SR), which is suitable for multi-ROI relationships, as the baseline for the previously established brain network containing multiple nodes (i.e., 101 nodes in the current study).^69^

To further validate the effectiveness of our proposed brain network in terms of its efficiency, and to provide evidence that the processing of social norms promotes the efficiency of punishment execution from the perspective of brain network efficiency, we conduct a complementary analysis. We selected typical brain network topology indicators for the network analysis, including small-world network characteristics and global efficiency. Integrating these topological indicators enables us to assess the speed and efficiency of information communication between brain network nodes. Following the recommendations of previous researchers,^121^ we examined the topological characteristics of brain networks. After excluding negative correlations, we calculated the weighted network based on graph theory using GRETNA.^121^ Ten sparsity levels from 0.04 to 0.94, which were computed through the function of [Rmax, Smin, Kmin] = gretna_get_rmax(rand(19,19)), were set to mitigate random effects while calculating brain network topological indicators (i.e., small-world network characteristics and global efficiency). Subsequently, the area under the curve, composed of ten sparsity levels, was used as the observation index for the two brain network metrics.
