# Supplementary material for: Neural architecture of social punishment: Insights from a queue-jumping scenario
Source: iScience. 2025 Feb 11;28(3):111988. doi: 10.1016/j.isci.2025.111988 (PMC11903947; doi:10.1016/j.isci.2025.111988)
Supplement: Document S1. Figures S1 and S2, Tables S1–S16, and Methods S1–S5 [file mmc1.pdf]

**iScience, Volume *28***

## Supplemental information

# Neural architecture of social punishment: Insights from a queue-jumping scenario

**Jiajia Zhu (朱佳佳), Xiruo Zhang (张熙若), Xiaotao Liu (刘晓涛), and Yan Mu (穆妍)**

**Supplemental Information**

This file includes:

- Tables S1 to S16
- Figures S1 to S2
- Supplementary References 1 to 17

**Methods S1: Conversion between 4-point and 2-point punishment rating (logistic regression), related to figure 1**

Because punishment decision in real-life is a binary choice, we combined the four-point score into a dichotomous choice of punishment or not (for a question about whether there is a difference between the four-point score and the dichotomous choice, see our Supplemental Study 4; for specific results, see the "Ecological validity and generalizability of the queue-jumping paradigm" section in the main text, and for specific research methods, see the Method details section in the main text). Since logistic regression is applicable to dichotomous results, logistic regression was employed to further elucidate how violation severity, psychological costs, and resource scarcity would jointly modulate punishment tendency. We introduced the three factors and all possible interaction terms into a logistic regression using the R package *caret* [1]. Ratings classified as "highly unlikely" and "mildly unlikely" to punish were categorized as low punishment tendency, while ratings classified as "mildly likely" and "highly likely" to punish were categorized as high punishment tendency. We randomly allocated 70% of responses as the training set and retained 30% as the test set. For model construction, we used leave-one-out cross-validation (LOOCV). The model demonstrated a prediction accuracy of 69.54% on the test set (AUC = 0.69, AIC = 3640.10, $\beta_{\text{violation severity}} = 0.71$, $p < 0.001$; $\beta_{\text{psychological costs}} = 0.67$, $p < 0.001$; $\beta_{\text{resource scarcity}} = 0.45$, $p < 0.001$; $\beta_{\text{psychological costs × violation severity}} = -0.31$, $p < 0.001$; $\beta_{\text{violation severity × resource scarcity}} = 0.10$, $p = 0.02$; $\beta_{\text{psychological costs × resource scarcity}} = 0.04$, $p = 0.32$; $\beta_{\text{violation severity × psychological costs × resource scarcity}} = 0.08$, $p = 0.07$). The interaction between the degree of violation and resource scarcity suggests that the shortage of resources amplifies participants' sensitivity to violations.

**Methods S2: Ordered probit regression, related to figure 1**

Given that linear models may not fully account for situations, we used ordered probit regression to analyze how violation severity, psychological costs, and resource scarcity influence punishment tendency using STATA 17 (https://www.stata.com/stata17). We assumed that $Y^*$ [$Y^* \sim N$ (0-1)], representing the participants' latent punishment possibility, was a linear function of the explanatory variable X: $Y^* = X \cdot \beta + \varepsilon$, where β represents the coefficient vector, and the residuals follow a standard normal distribution. We defined $y_i$ as the observed value of Y*, where i = 1, 2, 3, 4. And $\mu_i$ was defined as the i segmented points on the normal distribution of Y*, serving as the psychological thresholds for distinct punishment tendencies. Punishment tendency was obtained based on the relative size of the real value of the Y* and $\mu_i$. For example, if a participant' s real punishment tendency meets $Y^*_i \le \mu_1$, the participant chooses Y = 1. Therefore, Y* complies with $Y^* = \beta_{\text{violation severity}} X_1 + \beta_{\text{psychological costs}} X_2 + \beta_{\text{resource scarcity}} X_3 + \varepsilon$, $\varepsilon \sim N$ (0-1), where:

$y_i = 1, Y^*_i \le \mu_1$

$y_i = 2, \mu 1 \le Y^*_i \le \mu_2$

$y_i = 3, \mu 2 \le Y^*_i \le \mu_3$

$y_i = 3, \mu 3 \le Y^*_i$

$\Pr(Y=i) = F(\mu_i - (\beta_{\text{violation severity}} X_1 + \beta_{\text{psychological costs}} X_2 + \beta_{\text{resource scarcity}} X_3 + \varepsilon)) - F(\mu_{i-1} - (\beta_{\text{violation severity}} X_1 + \beta_{\text{psychological costs}} X_2 + \beta_{\text{resource scarcity}} + X_3 + \varepsilon))$.

Specifically, we introduced violation severity, psychological costs, resource scarcity, and four possible interaction terms into an ordered probit model. A Wald test of the interactions showed that violation severity significantly interplays with resource scarcity and psychological costs ($Chi^2_{\text{violation severity × psychological costs}}$ = 156.11, $p$ < 0.001; $Chi^2_{\text{violation severity × resource scarcity}}$ = 18.50, $p$ < 0.001; $Chi^2_{\text{psychological costs × resource scarcity}}$ = 3.90, $p$ = 0.05; $Chi^2_{\text{violation severity × resource scarcity × psychological costs}}$ = 0.22, $p$ = 0.63). Therefore, the final model incorporated the three factors along with the significant interactions mentioned above. It revealed that all the three factors exhibited marginal increasing effects on punishment tendency. Regarding violation severity, as the degree of violation escalated, the impact on punishment tendency intensified. Specifically, with each unit increase in violation severity, there was a 12% rise in the likelihood of choosing the highest punishment tendency. In addition, we found a similar marginally increasing effect of psychological costs on punishment tendency, with each unit increasing in psychological costs leading to a 10% increase in choosing the highest punishment tendency. Furthermore, the main effect of resource scarcity revealed that with each unit's increased resource insufficiency, the possibility of reporting the highest punishment tendency surged by 8%. This suggests that resource threat in the environment boosted individuals' punishment tendencies.

The current results have shown that as long as the violation severity was high in the queue-jumping situation, participants would exhibit a higher tendency to punish, regardless of the level of psychological costs. However, when the severity of the violation was low, participants showed a higher tendency to punish when the levels of psychological costs were high. The findings showed that when violation severity is high, the interaction between violation severity and psychological costs positively influences individuals' punishment tendencies. However, when violation severity is low, the interaction depends on the level of psychological costs, with a negative effect observed at lower levels of psychological costs. This variability results in an overall negative interaction effect.

**Methods S3: Decision tree analysis, related to figure 1**

Considering the potential interactions between resources and other variables, we conducted decision tree analyses at two levels of resource scarcity. Specifically, we conducted two additional decision tree analyses under conditions of resource sufficiency (prediction accuracy = 0.62, 95% CI = [0.59, 0.66) and insufficiency (prediction accuracy = 0.77, 95% CI = [0.73, 0.80). The results revealed that the influence of violation severity outweighed the impact of psychological costs regardless of resource levels in the queue-jumping context.

**Methods S4: Punishment tendency and reaction time, related to figure 1**

We performed a series of regression models to examine the relationship between punishment tendency and reaction times (RTs).s Participants' punishment tendency was assessed on a 4-point ordinal scale ranging from 'highly unlikely to punish' to 'highly likely to punish', which was suitable for ordered regression models [2]. To simultaneously account for the consistent relationship (i.e., fixed effects) between punishment tendency and RT as well as the random factors introduced by individual differences (i.e., random effects, [3]), we employed multilevel logit ordinal regression models using Bayesian statistical methods by the 'brms' package in R [4–8], which offer greater flexibility, interpretability, and robustness for addressing this question.

We used the cumulative logit link as the likelihood function, given its alignment with the logistic distribution of punishment ratings and their ordinal nature. Consistent with Reimer et al. (2021) [9], we applied conservative, weakly informative priors to mitigate bias and accurately shape the posterior distribution [10]. In the population-level (fixed effect) analysis, the estimated effect of RT on punishment tendency is -0.52, suggesting that with each unit increase in RT, there is a corresponding decrease in the log odds of moving to a higher rating on punishment tendency. We further investigated the marginal effects (i.e., the average effect of changes in predictors on the changes in the probability of outcomes [11]) to elucidate the influence of RTs on the predicted probabilities of punishment ratings. The results revealed that prior to the first quantile of RTs (0.39), participants tended to report higher levels of punishment tendency. However, this inclination gradually declined after reaching the median RT (0.52), with the probability increasing for lower levels of punishment tendency.

**Methods S5: The consideration of collective interests is the primary driver of social punishment, related to STAR Methods**

We explored the brain regions associated with resource scarcity to reveal how this contextual factor affects participants' punishment tendencies. The GLM analysis revealed that the left temporo-parietal junction (TPJ) [x = -57, y = -63, z = 27, $p_{FWE} = 0.002$, $t = 3.06$, k = 38, angular gyrus part, small volume correction] exhibited greater activation in the contrast between insufficient and sufficient conditions. TPJ is known to be involved in intention inference and theory of mind, suggesting that participants might make more inferences about the intentions of the queue jumper or others in the queue when resources are insufficient [12,13].

MVPA results demonstrated that neural activity patterns of the right precentral gyrus and the right caudate nucleus could distinguish between insufficient and sufficient conditions (> 50%) [precentral gyrus: x = 30, y = -18, z = 48, $p_{FWE} < 0.001$, t = 5.57, $Z_k = 4.96$; caudate: x = 15, y = -9, z = 27, $p_{FWE} < 0.001$, t = 5.76, $Z_k = 5.10$; brain regions reaching cluster-level significance at $p < 0.05$ (the FWE corrected values) with a cluster-forming threshold of $p < 0.001$ and a cluster size k ≥ 30 voxels were reported]. The right caudate nucleus is involved in norm compliance and assessing willingness to punish [14,15]. The finding suggests that participants exhibit a stronger desire to comply with social norms when resources are relatively insufficient.

**Methods S5: Punishment tendency and reaction time, related to STAR Methods**

***Relative Position.*** In the context of queue dynamics, the relative position of queue jumpers in relation to participants is a critical variable that deserves attention. To address this, we employed two distinct measures to mitigate the potential impact of the queue jumpers' positions within the queue on the outcomes. First, to avoid potential mutual interference, we conducted a linear mixed-effects modeling (LMM) regression using relative position (i.e., front vs. behind: whether the queue-jumper was in front of or behind the participant) as the predictor to examine its impact on punishment tendency. The linear mixed-effects (LME) models using the R package lme4 [16]. The regression analysis indicated that participants were more likely to punish when the queue-jumpers were in front of them, rather than behind them ($\beta_{\text{relative position}} = 0.53$, $p < 0.001$). Additionally, we tested the brain activity associated with relative position. In this study, we first observed activation of the left PCC, a brain region

associated with processing social punishment under strong social norm violations, when comparing scenarios where queue jumpers were in front versus behind. Additionally, we found hippocampal activation, a region involved in processing contextual information about transgressors in social decision-making [17]. This indicates that the hippocampus plays a role in encoding and integrating personalized context during social interactions involving norm violations. Notably, no significant activation was found when comparing queue jumpers positioned at the back versus the front (see Table S16).

In order to ensure the balance of conditions, that is, the level of violation severity and psychological costs are the same as six levels, we set the positions of the queue jumpers in the line (i.e., the front, middle, and back part of the queue). To verify whether these queue-jumping situations occur in daily life, that is, whether they have ecological validity, in Supplementary Studies 2 and 3, we asked participants to recall cases of queue jumping that they had experienced and to report the position of the queue jumpers in the queue (i.e., the front, middle, and back part of the queue). In Supplementary Studies 2 and 3, 17.00% and 14.74% of participants, respectively, reported queue jumpers appear at the back of the queue. This indicates that the setting of queue-jumpers appearing at the back of the queue (though less frequently) remains in line with real-life situations (middle of the queue: 23.00% in Study 2, 21.58% in Study 3; front of the queue: 56.50% in Study 2, 63.68% in Study 3).

**Table S1. Post hoc comparisons of violation severity and psychological costs across different levels**

| Comparisons between levels | | Mean difference | SE | t |
|---|---|---|---|---|
| ***1 = low to 6= high*** | | | | |
| ***Violation severity*** | | | | |
| 1 | 2 | -0.20 | 0.04 | $-4.90^{***}$ |
| | 3 | -0.40 | 0.05 | $-7.74^{***}$ |
| | 4 | -0.57 | 0.06 | $-9.91^{***}$ |
| | 5 | -0.81 | 0.08 | $-10.50^{***}$ |
| | 6 | -1.01 | 0.10 | $-10.53^{***}$ |
| 2 | 3 | -0.20 | 0.04 | $-4.62^{***}$ |
| | 4 | -0.38 | 0.05 | $-7.58^{***}$ |
| | 5 | -0.61 | 0.07 | $-8.86^{***}$ |
| | 6 | -0.82 | 0.09 | $-9.04^{***}$ |
| 3 | 4 | -0.17 | 0.03 | $-6.27^{***}$ |
| | 5 | -0.41 | 0.05 | $-8.61^{***}$ |
| | 6 | -0.61 | 0.07 | $-8.65^{***}$ |
| 4 | 5 | -0.23 | 0.04 | $-6.21^{***}$ |
| | 6 | -0.44 | 0.06 | $-7.03^{***}$ |
| 5 | 6 | -0.21 | 0.04 | $-5.26^{***}$ |
| ***Psychological costs*** | | | | |
| 1 | 2 | -0.22 | 0.04 | $-5.85^{***}$ |
| | 3 | -0.51 | 0.05 | $-9.48^{***}$ |
| | 4 | -0.64 | 0.07 | $-8.98^{***}$ |
| | 5 | -0.78 | 0.08 | $-10.32^{***}$ |
| | 6 | -0.82 | 0.10 | $-8.04^{***}$ |
| 2 | 3 | -0.29 | 0.04 | $-6.75^{***}$ |
| | 4 | -0.42 | 0.06 | $-7.50^{***}$ |
| | 5 | -0.55 | 0.07 | $-8.26^{***}$ |
| | 6 | -0.60 | 0.09 | $-6.57^{***}$ |
| 3 | 4 | -0.13 | 0.04 | $-3.46^{**}$ |
| | 5 | -0.26 | 0.06 | $-4.15^{***}$ |
| | 6 | -0.31 | 0.09 | $-3.32^{*}$ |
| 4 | 5 | -0.14 | 0.06 | -2.28 |
| | 6 | -0.18 | 0.09 | -2.15 |
| 5 | 6 | -0.05 | 0.06 | -0.86 |

***Note.*** SE: standard error. $^{*}p_{tukey} < 0.05$. $^{**}p_{tukey} < 0.01$. $^{***}p_{tukey} < 0.001$. Tukey: Tukey's post hoc test. The numbers 1 to 6 represent six levels of violation severity and psychological costs, with larger values indicating higher levels of norm violation / psychological costs.

**Table S2. Ordered probit estimates of the impacts of violation severity, psychological costs, and resource scarcity on punishment tendency.**

| Independent variables | Estimation | | | | Marginal effects | | | |
|---|---|---|---|---|---|---|---|---|
| | Coefficient (Standard error) | z | 95% confidence interval | | Punishment tendency dy/dx (p-value) | | | |
| | | | lower | Upper | 1 | 2 | 3 | 4 |
| Violation severity | $0.40^{***}$ (0.02) | 23.52 | 0.37 | 0.43 | $-0.09^{***}$ | $-0.04^{***}$ | $0.02^{***}$ | $0.12^{***}$ |
| Psychological costs | $0.35^{***}$ (0.02) | 20.71 | 0.32 | 0.38 | $-0.08^{***}$ | $-0.03^{***}$ | $0.01^{***}$ | $0.10^{***}$ |
| Resource scarcity | $0.27^{***}$ (0.02) | 16.28 | 0.24 | 0.30 | $-0.06^{***}$ | $-0.03^{***}$ | $0.01^{***}$ | $0.08^{***}$ |
| Violation severity × psychological costs | $-0.21^{***}$ (0.02) | -12.57 | -0.25 | -0.18 | $0.05^{***}$ | $0.02^{***}$ | $-0.01^{***}$ | $-0.06^{***}$ |
| Violation severity × resource scarcity | $0.07^{***}$ (0.02) | 4.33 | 0.04 | 0.11 | $-0.02^{***}$ | $-0.01^{***}$ | $0.00^{***}$ | $0.02^{***}$ |
| LR $Chi^2$ | 1314.25 | | | | | | | |
| Pseudo $R^2$ | 0.11 | | | | | | | |
| Log-likelihood | -5577.37 | | | | | | | |

*Note.* $^{***}p < 0.001$.

**Table S3. A summary of the ordinal logistic regression model on punishment tendency and reaction time (RT)**

| | Formula | Estimate | Est.Error | l-95% CI | u-95% CI | Rhat | Bulk ESS | Tail ESS |
|---|---|---|---|---|---|---|---|---|
| ***Population-level (fixed intercept)*** | | | | | | | | |
| | Punishment tendency ~ RT | | | | | | | |
| Intercept [1] | | -1.76 | 0.12 | -2.01 | -1.54 | 1 | 701 | 1300 |
| Intercept [2] | | -0.46 | 0.12 | -0.71 | -0.24 | 1 | 702 | 1202 |
| Intercept [3] | | 0.66 | 0.12 | 0.43 | 0.89 | 1 | 702 | 1126 |
| RT | | -0.52 | 0.11 | -0.74 | -0.3 | 1 | 4390 | 3236 |
| ***Population-Level Controlling for individual factors (random intercept)*** | | | | | | | | |
| | Punishment tendency ~ RT + (1\|participant) | | | | | | | |
| SD (Intercept) | | 0.70 | 0.08 | 0.57 | 0.88 | 1.00 | 767 | 1572 |

***Note.*** The intercept represents the log odds, which refer to the logarithm of the ratio between the probability of an event occurring and the probability of it not occurring. Positive log-odds values indicate a greater probability of favoring the lower ratings in each comparison, while negative log-odds values indicate a greater probability of favoring the higher ratings. Intercept [1]: The log-odds of choosing rating 2 (mildly unlikely to punish) and ratings below rating 2. Intercept [2]: The log-odds of choosing rating 3 (mildly likely to punish) and ratings below rating 3. Intercept [3]: The log-odds of choosing rating 4 (highly likely to punish) and ratings below rating 4. The Rhat value of 1.00 suggests excellent convergence. At convergence, Rhat should be 1 and not more than 1.1 [6].

SD: standard deviation; ESS (Effective Sample Size) is the effective number of samples, estimated from the samples obtained by the MCMC algorithm, that are both valid and independent. A higher ESS indicates a larger number of samples that can be effectively used to estimate the posterior distribution. Bulk-ESS represents the effective sample size of the main body of the posterior distribution, while Tail-ESS represents the effective sample size at both ends of the posterior distribution. The higher the ESS, the better; a rank-normalized ESS greater than 400 is typically sufficient to get a stable estimate of the Monte Carlo standard error.

**Table S4. Brain activation of the contrasts between different levels of violation severity**

| | MNI coordinates | | | | |
|---|---|---|---|---|---|
| | x | y | z | Voxel size | Max t-value |
| **Violation severity** | | | | | |
| ***High > low violation*** | | | | | |
| Fusiform gyrus (left) | -21 | -90 | -18 | 135 | $5.67^{*}$ |
| Inferior occipital gyrus (right) | 33 | -96 | -6 | 144 | $5.53^{*}$ |
| Post cingulate cortex (right) | 9 | -42 | 12 | 280 | $4.31^{**}$ |
| Dorsolateral prefrontal cortex (left)[1] | -24 | 42 | 45 | 33 | $3.75^{**}$ |
| ***Low > high violation*** | | | | | |
| Lingual (left) | -6 | -75 | -6 | 1640 | $8.58^{***}$ |
| Occipital pole (left) | -9 | -99 | 12 | | $7.64^{***}$ |
| Lingual (left) | 3 | -75 | -3 | | $8.31^{***}$ |

***Note.*** Brain regions reaching cluster-level significance at $p < 0.05$ (the FWE corrected values) with a cluster-forming threshold of $p = 0.001$ and a cluster size $k \geq 30$ voxels were reported. $^{*}p_{FWE} < 0.05$, $^{**}p_{FWE} < 0.01$, $^{***}p_{FWE} < 0.001$. 1 small volume correction.

**Table S5. MVPA results of the contrasts between high and low levels of violation severity**

| | MNI coordinates | | | | |
|---|---|---|---|---|---|
| | x | y | z | $Z_E$ | Max t-value |
| Cerebellum (left) | -9 | -66 | -21 | 3.93 | 4.23*** |
| Fusiform gyrus (left) | -24 | -75 | -9 | 3.65 | 3.65*** |
| Occipital pole (left) | -18 | -93 | -9 | 3.43 | 3.63*** |

***Note.*** Brain regions reaching cluster-level significance at $p < 0.05$ (the FWE corrected values) with a cluster-forming threshold of $p = 0.001$ and a cluster size $k \geq 30$ voxels were reported. $^{***}p_{FWE} < 0.001$.

**Table S6. Brain activation of the contrasts between different levels of psychological costs**

| | MNI coordinates | | | | |
|---|---|---|---|---|---|
| | x | y | z | Voxel size | Max t-value |
| **Personal costs** | | | | | |
| ***High > low personal costs*** | | | | | |
| Middle cingulate cortex (left) | -3 | -39 | 33 | 469 | 4.95*** |
| Dorsolateral prefrontal cortex (left) | -21 | 36 | 54 | 160 | 4.83* |
| Ventromedial prefrontal cortex (left) | -12 | 57 | 15 | 136 | 4.37* |
| ***High < low personal costs*** | No significant results | | | | |

***Note.*** Brain regions reaching cluster-level significance at $p < 0.05$ (the FWE corrected values) with a cluster-forming threshold of $p = 0.001$ and a cluster size $k \geq 30$ voxels were reported. $^{*}p_{FWE} < 0.05$, $^{***}p_{FWE} < 0.001$.

**Table S7. A summary of gPPI results of different contrasting conditions**

| Seed | Contrasting conditions | gPPI region | MNI coordinates | | | | | Max |
|---|---|---|---|---|---|---|---|---|
| | | | x | y | z | $K_E$ | $p_{FWE\ corr.}$ | t-value |
| dlPFC (left) | High > low violation | dorsal PCC (left) | -15 | -54 | 42 | 256 | 0.05 | 4.34 |
| dlPFC (left) | Low > high costs | Frontal pole[1](left) | -15 | 60 | 15 | 50 | 0.01 | 4.37 |
| | | dorsal ACC[1](left) | -9 | 30 | 27 | 55 | 0.01 | 4.13 |
| vmPFC (left) | High > low costs | IFG[1] (right) | 24 | 39 | -3 | 38 | 0.02 | 4.07 |
| | | OFC[1](right) | 18 | 30 | -9 | 35 | 0.02 | 3.28 |
| | Low > high costs | dorsal ACC[1] (right) | 18 | 30 | 33 | 34 | 0.02 | 3.6 |
| dlPFC (left) | Low > high costs | Frontal pole[1] (right) | -18 | 51 | 21 | 46 | 0.01 | 3.98 |

***Note.*** Brain regions reaching cluster-level significance at $p < 0.05$ (the FWE corrected values) with a cluster-forming threshold of $p = 0.005$ and a cluster size k ≥ 30 voxels were reported. dlPFC: dorsolateral prefrontal cortex; vmPFC: ventromedial prefrontal cortex; PCC: posterior cingulate cortex; ACC: anterior cingulate cortex; IFG: inferior frontal gyrus; OFC: orbital frontal cortex. [1]: small volume correction with 8-mm-radius ROI (region of interests).

**Table S8. A summary of the ROI nodes for constructing the punishment network**

| ROI | MNI coordinates | | | Analysis |
|---|---|---|---|---|
| | x | y | z | |
| Fusiform_L | -21 | -90 | -18 | |
| IOG_R | 33 | -96 | -6 | |
| PCC_R | 9 | -42 | 12 | GLM activation in the contrast between high and low violation |
| dlPFC_L | -24 | 42 | 45 | |
| SOG_L | -9 | -99 | 12 | |
| Cerebellum_L | -9 | -66 | -21 | |
| Fusiform _L | -24 | -75 | -9 | MVPA activation in the contrast between high and low violation |
| Occipital pole_L | -18 | -93 | -9 | |
| dPCC_L | -15 | -54 | 42 | gPPI connection with dlPFC in the contrast between high and low violation |
| MCC_L | -3 | -39 | 33 | |
| vmPFC_L | -12 | 57 | 15 | GLM activation in the contrast between high and low costs |
| dlPFC_L | -21 | 36 | 54 | |
| dACC_L | -9 | 30 | 27 | gPPI connection with dlPFC in the contrast between low and high costs |
| FP_L | -15 | 60 | 15 | |
| IFG_R | 24 | 39 | -3 | gPPI connection with vmPFC in the contrast between high and low costs |
| OFC_R | 28 | 30 | -9 | |
| dACC_R | 18 | 30 | 3 | gPPI connection with vmPFC in the contrast between low and high costs |
| dlPFC_L | -18 | 33 | 54 | GLM activation in the contrast between high and low punishment tendency |
| MTG_L | -57 | -30 | -15 | GLM: F test across different punishment tendencies |

***Note.*** IOG: inferior occipital cortex; SOG: superior occipital cortex; dlPFC: dorsolateral prefrontal cortex; vmPFC: ventromedial prefrontal cortex; PCC: posterior cingulate cortex; dPCC: dorsal posterior cingulate cortex; MCC: middle cingulate cortex; ACC: anterior cingulate cortex; dACC: dorsal anterior cingulate cortex; IFG: inferior frontal gyrus; OFC: orbital frontal cortex; FP: frontal pole; MTG: middle temporal gyrus.

**Table S9. Mean and standard deviation (SD) of punishment tendency for the two punishment groups.**

| | | Punishment tendency (1 = very unlikely to punish, 4 = very likely to punish, ) | |
|---|---|---|---|
| Group | N | Mean | SD |
| High punishment group | 37 | 2.41 | 0.23 |
| Low punishment group | 22 | 3.05 | 0.33 |

***Note.*** Participants were categorized based on the mean value of punishment tendency. Those scoring above the mean were designated as the high punishment group, while those scoring below were labeled as the low punishment group.

**Table S10. The performance of different brain network connections in distinguishing high and low punishment tendency groups**

| | AUC | ACC | SEN | SPE | BAC |
|---|---|---|---|---|---|
| FC (task) | 0.51 | 45.76% | 40.91% | 48.65% | 44.78% |
| **tHOFC (task)** | **0.58** | **61.02%** | 54.55% | 64.86% | 59.71% |
| FC (rest) | 0.40 | 39.66% | 31.82% | 44.44% | 38.13% |
| tHOFC (rest) | 0.48 | 51.72% | 40.91% | 58.33% | 49.62% |
| SR (task: previous network) | 0.51 | 54.24% | 22.73% | 72.97% | 47.85% |
| tHOFC (task: previous network) | 0.23 | 33.90% | 18.18% | 43.24% | 30.71% |
| **SR (resting: previous network)** | **0.39** | **60.34%** | 13.64% | 88.89% | 51.26% |
| tHOFC (resting: previous network) | 0.35 | 41.38% | 31.82% | 47.22% | 39.52% |

***Note.*** AUC: the area under ROC curve; ACC: classification accuracy; SEN: sensitivity, SPE: specificity, BAC: balance accuracy. FC: functional connectivity. tHOFC: topological high-order FC methods. SR: SR-based functional connectivity (SR: sparse representation or partial correlation).

**Table S11. Repeated measures ANOVA for the effects of violation severity and psychological costs on punishment tendency in the supplementary study**

| | Mean ± SE | Sum of Squares | Mean Square | F | $\eta^2_p$ |
|---|---|---|---|---|---|
| Violation severity | High: 4.82 ± 0.07<br>Low: 3.67 ± 0.05 | 329.02 | 329.02 | $422.64^{***}$ | 0.63 |
| Residual | | 191.51 | 0.78 | | |
| Psychological costs | High: 4.72 ± 0.07<br>Low: 3.77 ± 0.05 | 226.55 | 226.55 | $331.76^{***}$ | 0.57 |
| Residual | | 167.99 | 0.68 | | |
| Violation severity × psychological costs | Hv_Hc: 4.64 ± 0.09<br>Hv_Lc: 5.00 ± 0.06<br>Lv_Hc: 4.80 ± 0.06<br>Lv_Lc: 2.53 ± 0.07 | 427.99 | 427.99 | $621.37^{***}$ | 0.72 |
| Residual | | 169.44 | 0.69 | | |

***Note.*** SE: standard error. Hv: high violation; Lv: low violation; Hc:high costs; Lc: low costs. $^{*}p < 0.05$. $^{**}p < 0.01$. $^{***}p < 0.001$. N = 247.

**Table S12. Post hoc comparisons of violation severity and psychological costs on punishment tendency in the supplementary study**

| Comparison | Mean differences | SE | t |
|---|---|---|---|
| (High violation - high costs) vs. (high violation - low costs) | -0.36 | 0.07 | $-5.04^{***}$ |
| (High violation - high costs) vs. (low violation - high costs) | -0.16 | 0.08 | $-2.14^{*}$ |
| (High violation - high costs) vs. (low violation - low costs) | 2.11 | 0.1 | $20.99^{***}$ |
| (High violation - low costs) vs. (low violation - high costs) | 0.2 | 0.04 | $4.75^{***}$ |
| (High violation - low costs) vs. (low violation - low costs) | 2.47 | 0.08 | $31.60^{***}$ |
| (Low violation - high costs) vs. (low violation - low costs) | 2.27 | 0.08 | $29.24^{***}$ |

***Note.*** SE: standard error. $^{*}p < 0.05$. $^{**}p < 0.01$. $^{***}p < 0.001$.

**Table S13. A summary of severity and cost rankings for different queue-jumping scenarios in the supplementary study**

| **Post hoc comparison** (Mean of average raking ± SD) | | | | |
|---|---|---|---|---|
| **Violation severity** | | **Mean Difference** | **SE** | **t** |
| High (3.14 ± 1.27) | Medium (4.92 ± 0.54) | -1.78 | 0.10 | $-17.62^{***}$ |
| | Low (6.95 ± 1.13) | -3.81 | 0.15 | $-25.57^{***}$ |
| High | Low | -2.03 | 0.08 | $-25.79^{***}$ |
| **Psychological costs** | | | | |
| High (4.26 ± 0.94) | Medium (5.01 ± 0.51) | -0.75 | 0.08 | $-9.13^{***}$ |
| | Low (5.74 ± 0.79) | -1.48 | 0.11 | $-13.98^{***}$ |
| Medium | Low | -0.73 | 0.06 | $-12.19^{***}$ |

***Note.*** SE: standard error. SD: standard deviation. $^{***}p < 0.001$.

**Table S14. Post hoc comparisons between different punishment motivation items in the supplementary study**

| Comparisons | | | | |
|---|---|---|---|---|
| Punishment motivation items (Mean ± SD) | | Mean difference | SE | t |
| Increased waiting time (6.35 ± 0.79) | Social norm violation (6.29 ± 0.97) | 0.06 | 0.07 | 0.92 |
| | Violating personal norms (6.13 ± 1.04) | 0.22 | 0.07 | 3.12** |
| | Affecting ticket purchase (6.08± 1.00) | 0.27 | 0.07 | 3.69*** |
| | Causing chaos (6.01 ± 0.98) | 0.34 | 0.07 | 4.94*** |
| | Affecting others' interests (5.85 ± 1.41) | 0.49 | 0.07 | 7.03*** |
| Social norm violation | Violating personal norms | 0.16 | 0.07 | 2.34* |
| | Affecting ticket purchase | 0.21 | 0.08 | 2.49* |
| | Causing chaos | 0.28 | 0.07 | 3.87*** |
| | Affecting others' interests | 0.43 | 0.07 | 6.14*** |
| Violating personal norms | Affecting ticket purchase | 0.04 | 0.08 | 0.54 |
| | Causing chaos | 0.11 | 0.07 | 1.53 |
| | Affecting others' interests | 0.27 | 0.07 | 3.87*** |
| Affecting ticket purchase | Causing chaos | 0.07 | 0.08 | 0.85 |
| | Affecting others' interests | 0.23 | 0.09 | 2.57* |
| Causing chaos | Affecting others' interests | 0.16 | 0.07 | 2.15* |

***Note.*** SD: standard deviation. $^{*}p < 0.05$. $^{**}p < 0.01$. $^{***}p < 0.001$.

**Table S15. The correlations of punishment measures across contexts (i.e., queue-jumping, economic, criminal, social norm, daily)**

| | Descriptives | 1 | 2 | 3 | 4 | 5 | 6 | 7 | 8 |
|---|---|---|---|---|---|---|---|---|---|
| **1.Queue-jumping task** | 4.25 ± 0.84 | — | | | | | | | |
| 2. Criminal (intentional) | 6.42 ± 0.60 | 0.01 | — | | | | | | |
| 3. Criminal (unintentional) | 4.15 ± 1.27 | $0.20^{**}$ | -0.01 | — | | | | | |
| 4. Norm violation scenario (intentional) | 5.85 ± 0.82 | 0.11 | $0.25^{***}$ | -0.03 | — | | | | |
| 5. Norm violation scenario (unintentional) | 4.29 ± 1.08 | $0.16^{*}$ | 0.06 | $0.39^{***}$ | $0.45^{***}$ | — | | | |
| 6. Norm violation scenario (neutral) | 2.23 ± 0.86 | $0.18^{**}$ | $-0.14^{*}$ | $0.33^{***}$ | 0.03 | $0.27^{***}$ | — | | |
| 7. Economic paradigm | Punish: 27% | 0.01 | $0.13^{*}$ | -0.07 | $0.16^{*}$ | 0.00 | -0.11 | — | |
| 8. Daily life | 3.12 ± 0.84 | $0.31^{***}$ | -0.10 | $0.14^{*}$ | 0.03 | $0.15^{*}$ | $0.22^{***}$ | $0.13^{*}$ | — |

***Note.*** SD: standard deviation. Descriptive results including M ± SD (for queue jumping paradigm, criminal scenario, and social norm violation scenario) and the proportion of participants making punishment decisions (for economic paradigm). $^{*}p < 0.05$. $^{**}p < 0.01$. $^{***}p < 0.001$.

**Table S16. Brain activation of the main effect of relative anteroposterior position of queue jumpers and participants**

| | MNI Coordinates | | | | |
|---|---|---|---|---|---|
| | x | y | z | Voxel size | Max t-value |
| ***Front > Behind*** | | | | | |
| L PCC | -6 | -21 | 36 | 1010 | $6.11^{***}$ |
| L Second visual association cortex | -27 | -96 | -12 | 145 | $5.43^{*}$ |
| L Hippocampus | -30 | -18 | -21 | 153 | $5.41^{*}$ |
| ***Behind > Front*** | | | | | |

***Note.*** Brain regions reaching cluster-level significance at $p < 0.05$ (the FWE corrected values) with a cluster-forming threshold of $p = 0.001$ and a cluster size $k \geq 30$ voxels were reported. $^{*}p_{FWE} < 0.05$, $^{***}p_{FWE} < 0.001$.

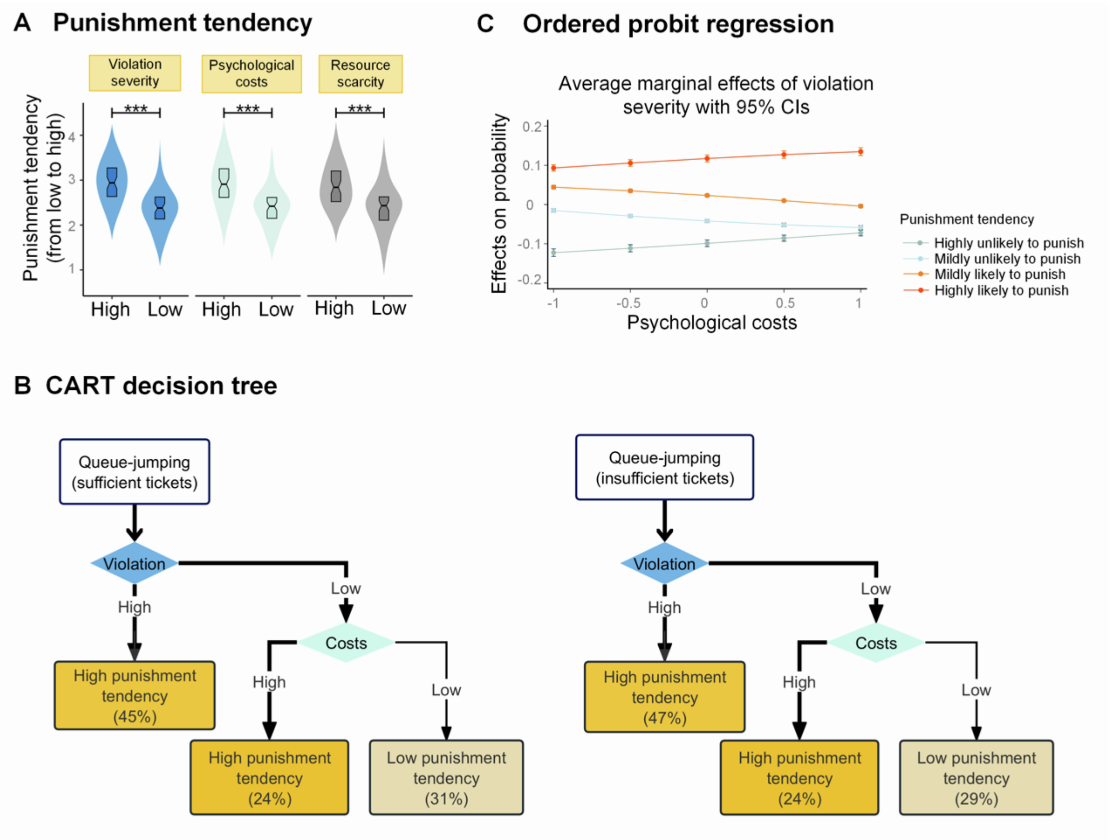


**Fig. S1. Behavioral results. (A) Verification of condition division.** For the levels of violation and psychological costs, we categorized the initial three levels as the low level of violations (or costs) and the subsequent three as the high level of violations (or costs). Paired sample t-tests showed that higher levels of violation ($t$ (58) = 10.28, $p$ < 0.001, Cohen's d = 1.34), psychological costs ($t$ (58) = 8.78, $p$ < 0.001, Cohen's d = 1.14), and resource scarcity ($t$ (58) = 6.93, $p$ < 0.001, Cohen's d = 0.92) activated an increased tendency among participants to administer punishment compared to lower levels of these conditions. In the boxplot, the upper edge represents the third quartile, while the lower edge indicates the first quartile. The horizontal line within the box denotes the median. **(B) CART decision tree.** The structure of decision tree underscores that participants prioritize the preservation of norms as their primary concern when administer punishment, followed by the protection of personal interests. Resource scarcity did not affect this cognitive pathway. Numbers in parentheses indicate the proportion of responses in the current path to the total. **(C) Ordered probit regression.** We calculated the marginal effects of violation severity across five levels of psychological costs (scaled data: -1, -0.5, 0, 0.5, 1). The results revealed that violation severity had marginal increasing effects on participants' highest punishment tendency (i.e., 4-point, *highly likely to punish*). Additionally, as the degree of psychological costs increased, there was a marginal diminishing effect of violation severity on participants' tendency to choose ambiguous punishment (i.e., 2-point, *mildly unlikely to punish*, and 3-point, *mildly likely to punish*). Data are represented as mean +/- SE.

***Note.*** $^{***}p$ < 0.001. SE: Standard Error.

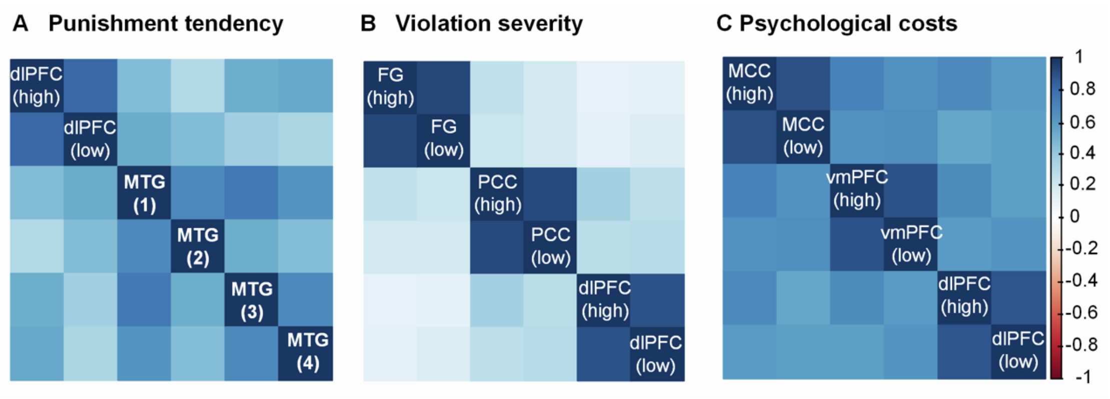


**Fig. S2. The correlations between the activities of distinct brain regions. (A) Correlations between brain regions associated with punishment tendency.** There were positive correlations between the intensities of the MTG and the anterior dLPFC. **(B) Correlations among brain regions associated with violation severity.** There were positive correlations among the intensities of brain regions associated with violation severity. **(C) Correlations among brain regions associated with psychological costs.** There were positive correlations among the intensities of brain regions related to psychological costs. ***Note.*** dlPFC: dorsolateral prefrontal cortex; MTG: middle temporal gyrus; vmPFC: ventromedial prefrontal cortex; PCC: posterior cingulate cortex; MCC: middle cingulate cortex. In Fig.S2A, *high* indicates high punishment tendency and *low* indicates low punishment tendency; 1: *highly unlikely to punish*, 2: *mildly unlikely to punish*, 3: *mildly likely to punish*, 4: *highly likely to punish*. In Fig.S2B, *high* indicates high violation severity and *low* indicates low violation severity. In Fig. S2C, *high* means high psychological costs and *low* means low psychological costs.

## References


1. Kuhn, M. (2008). Building Predictive Models in R Using the caret Package. J. Stat. Softw. *28*. https://doi.org/10.18637/jss.v028.i05.
2. Harrell , F.E. (2015). Regression Modeling Strategies: With Applications to Linear Models, Logistic and Ordinal Regression, and Survival Analysis (Springer International Publishing) https://doi.org/10.1007/978-3-319-19425-7.
3. Gardiner, J.C., Luo, Z., and Roman, L.A. (2009). Fixed effects, random effects and GEE: What are the differences? Stat. Med. *28*, 221–239. https://doi.org/10.1002/sim.3478.
4. Bürkner, P.-C. (2017). Advanced Bayesian Multilevel Modeling with the R Package brms. Preprint at arXiv, https://doi.org/10.48550/arXiv.1705.11123 https://doi.org/10.48550/arXiv.1705.11123.
5. Bürkner, P.-C. (2020). Bayesian Item Response Modeling in R with brms and Stan. Preprint at arXiv, https://doi.org/10.48550/arXiv.1905.09501 https://doi.org/10.48550/arXiv.1905.09501.
6. Bürkner, P.-C., and Vuorre, M. (2019). Ordinal Regression Models in Psychology: A Tutorial. Adv. Methods Pract. Psychol. Sci. *2*, 77–101. https://doi.org/10.1177/2515245918823199.
7. Carpenter, B., Gelman, A., Hoffman, M.D., Lee, D., Goodrich, B., Betancourt, M., Brubaker, M.A., Guo, J., Li, P., and Riddell, A. (2017). Stan: A Probabilistic Programming Language. J. Stat. Softw. *76*, 1. https://doi.org/10.18637/jss.v076.i01.
8. Bürkner, P.-C. (2017). brms: An R Package for Bayesian Multilevel Models Using Stan. J. Stat. Softw. *80*. https://doi.org/10.18637/jss.v080.i01.
9. Reimer, N.K., Love, A., Wölfer, R., and Hewstone, M. (2021). Building Social Cohesion Through Intergroup Contact: Evaluation of a Large-Scale Intervention to Improve Intergroup Relations Among Adolescents. J. Youth Adolesc. *50*, 1049–1067. https://doi.org/10.1007/s10964-021-01400-8.
10. Lemoine, N.P. (2019). Moving beyond noninformative priors: why and how to choose weakly informative priors in Bayesian analyses. Oikos *128*, 912–928. https://doi.org/10.1111/oik.05985.
11. Norton, E.C., Dowd, B.E., and Maciejewski, M.L. (2019). Marginal Effects—Quantifying the Effect of Changes in Risk Factors in Logistic Regression Models. JAMA *321*, 1304. https://doi.org/10.1001/jama.2019.1954.
12. Krueger, F., and Hoffman, M. (2016). The Emerging Neuroscience of Third-Party Punishment. Trends Neurosci. *39*, 499–501. https://doi.org/10.1016/j.tins.2016.06.004.
13. Feng, C., Yang, Q., Azem, L., Atanasova, K.M., Gu, R., Luo, W., Hoffman, M., Lis, S., and Krueger, F. (2022). An fMRI investigation of the intention-outcome interactions in second- and third-party punishment. Brain Imaging Behav. *16*, 715–727. https://doi.org/10.1007/s11682-021-00555-z.
14. de Quervain, D.J.-F., Fischbacher, U., Treyer, V., Schellhammer, M., Schnyder, U., Buck, A., and Fehr, E. (2004). The Neural Basis of Altruistic Punishment. Science *305*, 1254–1258. https://doi.org/10.1126/science.1100735.

15. Spitzer, M., Fischbacher, U., Herrnberger, B., Groen, G., and Fehr, E. (2007). The neural signature of social norm compliance. Neuron *56*, 185–196. https://doi.org/10.1016/j.neuron.2007.09.011.
16. Bates, D., Mächler, M., Bolker, B., and Walker, S. (2014). Fitting Linear Mixed-Effects Models using lme4. Preprint at arXiv, https://doi.org/10.48550/arXiv.1406.5823 https://doi.org/10.48550/arXiv.1406.5823.
17. FeldmanHall, O., Montez, D.F., Phelps, E.A., Davachi, L., and Murty, V.P. (2021). Hippocampus Guides Adaptive Learning during Dynamic Social Interactions. J. Neurosci. *41*, 1340–1348. https://doi.org/10.1523/JNEUROSCI.0873-20.2020.
